# Opposite Polarity Monospore Genome De Novo Sequencing and Comparative Analysis Reveal the Possible Heterothallic Life Cycle of *Morchella importuna*

**DOI:** 10.3390/ijms19092525

**Published:** 2018-08-25

**Authors:** Wei Liu, LianFu Chen, YingLi Cai, QianQian Zhang, YinBing Bian

**Affiliations:** 1 Institute of Applied Mycology, Plant Science and Technology College, Huazhong Agricultural University, Wuhan 430070, China; zhenpingliuwei@163.com (W.L.); chenllianfu@foxmail.com (L.C.); loveylcai@163.com (Y.C.); qzhang196@163.com (Q.Z.); 2 Key Laboratory of Agro-Microbial Resource Comprehensive Utilization, Ministry of Agriculture, Huazhong Agricultural University, Wuhan 430070, China

**Keywords:** *Morchella importuna*, mating type, transcriptome, heterothallism, gene expansion and contraction

## Abstract

*Morchella* is a popular edible fungus worldwide due to its rich nutrition and unique flavor. Many research efforts were made on the domestication and cultivation of *Morchella* all over the world. In recent years, the cultivation of *Morchella* was successfully commercialized in China. However, the biology is not well understood, which restricts the further development of the morel fungus cultivation industry. In this paper, we performed de novo sequencing and assembly of the genomes of two monospores with a different mating type (M04M24 and M04M26) isolated from the commercially cultivated strain M04. Gene annotation and comparative genome analysis were performed to study differences in CAZyme (Carbohydrate-active enzyme) enzyme content, transcription factors, duplicated sequences, structure of mating type sites, and differences at the gene and functional levels between the two monospore strains of *M. importuna*. Results showed that the de novo assembled haploid M04M24 and M04M26 genomes were 48.98 and 51.07 Mb, respectively. A complete fine physical map of *M. importuna* was obtained from genome coverage and gene completeness evaluation. A total of 10,852 and 10,902 common genes and 667 and 868 endemic genes were identified from the two monospore strains, respectively. The Gene Ontology (GO) and KAAS (KEGG Automatic Annotation Serve) enrichment analyses showed that the endemic genes performed different functions. The two monospore strains had 99.22% collinearity with each other, accompanied with certain position and rearrangement events. Analysis of complete mating-type loci revealed that the two monospore *M. importuna* strains contained an independent mating-type structure and remained conserved in sequence and location. The phylogenetic and divergence time of *M. importuna* was analyzed at the whole-genome level for the first time. The bifurcation time of morel and tuber was estimated to be 201.14 million years ago (Mya); the two monospore strains with a different mating type represented the evolution of different nuclei, and the single copy homologous genes between them were also different due to a genetic differentiation distance about 0.65 Mya. Compared with truffles, *M. importuna* had an extension of 28 clusters of orthologous genes (COGs) and a contraction of two COGs. The two different polar nuclei with different degrees of contraction and expansion suggested that they might have undergone different evolutionary processes. The different mating-type structures, together with the functional clustering and enrichment analysis results of the endemic genes of the two different polar nuclei, imply that *M. importuna* might be a heterothallic fungus and the interaction between the endemic genes may be necessary for its complete life history. Studies on the genome of *M. importuna* facilitate a better understanding of morel biology and evolution.

## 1. Introduction

The kingdom of fungi (Eumycota) is a group of eukaryotes present in almost all habitats, which not only play an important role in nature, but also affect human life in different ways [1]. The largest group among the fungi is the Ascomycetes, which consists of Saccharomycotina, Taphrinomycotina, and Pezizomycotina. The former (Saccharomycotina and Taphrinomycotina) contain many unicellular species or dimorphic fungi, whereas Pezizomycotina are generally composed of filamentous fungi capable of producing highly differentiated multicellular structures, with the most basal groups being Pezizomycetes and Orbiliomycetes, which form open fruiting bodies (apothecia or ascocarps) with exposed meiosporangia (asci) for the development of ascospores [1,2].

True morels (*Morchella* spp.), belonging to Ascomycota, are consumed and appreciated worldwide due to their savory flavor and multiple bioactivities, including anti-oxidative, anti-inflammatory, anti-microbial, immunostimulatory, and anti-tumor properties [3,4]. Therefore, the domestication and cultivation of *Morchella* aroused the attention of mushroom enthusiasts worldwide [5,6,7]. The earliest domestication of *Morchella* can be dated back to 1882. However, until 1982, the American scientist, Ower realized the indoor cultivation of morel, then carried out industrialized indoor production; however, the indoor cultivation did not last a long time due to a lack of basic biological knowledge and other unknown reasons [3,5,6,8,9,10]. On the basis of Ower’s pioneering study, Israeli scientists carried out the indoor soil-free cultivation, and performed some cytological research on the development of the primordium of *M. rufobrunnea* using the experimental materials obtained from this cultivation; however, no commercial cultivation was reported [7,11]. Fortunately, Ower’s work gradually embarked on the road of commercialization in China. In the long period of development, Chinese morel cultivation made a breakthrough in the technology of morel spawns and the feeding of foreign nutrition bags to realize commercialized cultivation in outdoor fields [3,12]. With this technology, more than 1 kg of fresh mushrooms can be produced per square meter under the experimental conditions or in some incidental cases [3]. In 2017, the area of morel cultivation reached 4666 hectares in China [13]. However, commercialized cultivation was compromised due to a shortage of basic biological information about morel, such as the strain polarity and ploidy, life cycle, nutrition metabolism, aging, and degradation, leading to an average production of less than 1500 kg/ha (fresh weight) and making more than 70% of practitioners unable to earn a stable profit [12,13].

Early *Morchella* research focused on the types of nutrition [14,15], physiological metabolism [16,17], development [11,18,19,20,21], nutritional value [22,23,24], and population genetics [25]. In recent years, researchers paid more attention to the classification and genetic field [3,4,26,27]. The complete life cycle of morel could not be accomplished under laboratory conditions, which is the main difficulty in solving many genetic problems about morel [3,6]. Multigenic phylogeny is widely used in the classification of *Morchella* spp., and until now, there are about 69 phylogenetic species of *Morchella* sp., mainly in the north temperate zone, including Europe, Asia, and North America [28,29,30,31,32,33]. In recent years, some progress was achieved in the research on primordium development, aging, sclerotium development, and mating type of *Morchella* [4,26,27]. Under the controlled laboratory conditions, Masaphy S. (2005) examined the external morphological changes during initiation and early stages of fruit-body development of a *Morchella* sp. using scanning electron microscopy [11]. Additionally, Masaphy S. (2010) reported, for the first time, the successful fruiting-body production of *M. rufobrunnea* under controlled conditions, and described in detail its entire life cycle and the five morphological developmental stages, including sclerotium formation, germination, asexual spore formation, formation of initial knots, and development of the fruiting body [7]. A variety of *Morchella*, including *Morchella elata*, showed signs of developmental aging [34]. Aging strains cannot be used for commercial cultivation due to their difference from normal strains in growth speed, sclerotial development, mycelial morphology, pigment production, and subcellular structure [3,34]. Sclerotium is an important link in the life history of *Morchella* [12]. Early cultivation is mainly based on the direct induction of mushroom after sclerotial culture [14]. Specific genes were studied during the development of sclerotia, and their development involves the processes of autophagy, apoptosis, and energy accumulation as oil droplets [12]. Previous studies provided cytological and molecular biological evidence for characteristics of the homokaryon of monospores of *Morchella importuna*, as well as population genetic evidence for heterothallic characteristics of the ascocarp of morel; however, there is no direct evidence for hybridization fruiting [4,26,27].

Genomics research is a window to understanding a specific species. Many species in the Ascomycota family were sequenced and analyzed [1]. The truffles genome facilitates the study of the energy metabolism, species evolution, population genetics, and mating structure [35]. The genome sequence of *Sclerotinia sclerotiorum* deepened our understanding of its life history and infection process [36]. For *Pyronema confluens*, a saprobic Pezizomycete with a typical apothecium as a fruiting body, genome data facilitated the understanding of its fungal evolution in the basal filamentous ascomycetes [1]. *Morchella*, belonging to the family Morchaceae, includes four hypogeous genera, *Kalapuya*, *Fischerula*, *Leucangium*, and *Imaia*, and three genera with ascocarps growing on the ground *Disciotis*, *Morchella*, and *Verpa*, with *Morchella* being the most famous species among them [37]. To our best knowledge, there is no report on any genome research of *Morchella* (except sequencing work which was carried out by the 1000 Genomes Project on *Morchella conia*, *Morchella importuna*, and *Gyromitra esculenta*, for which the results of sequencing and assembly were uploaded, but without any in-depth analysis).

In this paper, two different polar monospore strains of *M. importuna* were used for deep de novo genome sequencing, assembly, and functional annotation. A fine and complete genome of *M. importuna* was obtained for the first time, and a comparative genome analysis was carried out between the two monospore strains with opposite polarity. The polar nuclei were analyzed in terms of genome sequence, gene structure, and evolution difference at the nuclear level, hoping to deepen the understanding of morel biology and evolution and to facilitate the molecular genetics analysis and breeding of *M. importuna*.

## 2. Results

### 2.1. Sequencing Strategy and Data Statistics

In this study, genome-wide sequencing was performed for the two different mating genotypes of monospores M04M24 and M04M26 isolated from the commercial strain and parent strain, *M. importuna* M04. The M04M24 and M04M26 strains were identified as the MAT1-2 and MAT1-1 mating type, respectively, based on Illumina Hiseq 4000 sequencing with the third-generation Pacbio sequencing technology, using a 20-kb library of insert fragments. A total of seven different insert libraries were constructed. After filtering, the low-quality, duplication, joint-sequence, and short reads were removed from the raw data, and a total of 27.6 Gb of clean data were obtained (Table S1). 

The genomic characteristics of the M04M26 strain were assessed with paired-end library data using Kmer Spectrum Analysis. Specifically, the genome size of the M04M26 strain was estimated to be 54.15 Mb (The M04M24 strain was also estimated to be 54.74 Mb, which was almost the same as the M04M26 strain), with non-repetitive sequences estimated to be 39.36 Mb (72.7%), and repeat sequences (sequence length greater than 25 bp with a repetition of more than one) estimated to be 14.78 Mb (27.30%) of the whole genome.

### 2.2. De Novo Genomic Assembly

Due to the unavailability of reference information about the *Morchella* genome, a de novo assembly strategy was used to assemble the *M. importuna* genome. In this paper, de novo genomes of the two monospore strains M04M24 and M04M26 with a different mating type were finally obtained, with scaffold numbers of 394 and 106, contig numbers of 763 and 111, scaffold N50 sizes of 653.8 and 978.7 kb, contig N50 sizes of 198.4 and 951.6 kb, and longest sequence lengths of 2.65 and 3.55 Mb, while the actual assemblies were 48.98 and 51.07 Mb for the M04M24 and M04M26 strains, respectively. The genome completeness in terms of conserved proteins was estimated to be 90.45% and 94.31%, respectively. The assembly results of the two single-spore genomes showed that the percentages of GC content in the two genomes tended to be consistent with each other (~47.3%) (Table 1). The final de novo genomic assembly results were submitted to the National Center for Biotechnology Information (NCBI), with public accession numbers QORM00000000 for monospore M04M24 and QOKS00000000 for monospore M04M26.

### 2.3. Protein Prediction and Functional Annotation

The protein-encoding genes in the whole genomes of the two monospores were predicted by the alignment of homologous proteins and the transcriptional data, and ab initio predictions [38]. A total of 11,519 and 11,770 protein-encoding genes were predicted for the two monospores (M04M24 and M04M26, respectively) with different mating type. The number of common genes was 10,852 and 10,902 for the two different polar monospore strains, with 667 endemic genes in M04M24 and 868 endemic genes in M04M26, and the total number of genes for *M. importuna* was estimated to be between 11,569 and 11,720. The genomic structures of the two monospore strains were analyzed, with gene median lengths of 1304 bp (M04M24) and 1282 bp (M04M26), intergenic median lengths of 1614 bp (M04M24) and 1634 bp (M04M26), complementary DNA (cDNA) median lengths of 1038 bp (M04M26) and 1053 bp (M04M24), average exon numbers of 3.96 and 3.99, and an equal single-intron median length of 66 bp. These findings are similar to those in *Tuber melanosporum*, *Pyronema confluens*, *Aspergillus oryzae*, and *Sordaria macrospora* [1,35,39,40]. The average lengths of total protein were 449.07 amino acids (aa) (M04M24) and 442.37 aa (M04M26) (Table S2). The longest proteins were MIM04M24Gene01232 (M04M24) and MIM04M26Gene03930 (M04M26), with 7124 and 7125 amino acids, respectively. Functional annotation and homology analysis showed that the two genes were identical, and functionally annotated as putative involucrin repeat protein (Expect (E)-value 6.16852 × 10^−104^ with GI|615406126|ref|XP_007581915.1|). The completeness of the genome of the eukaryotes was evaluated using the BUSCO software, with 289 and 287 conserved proteins predicted out of the 290 reference-conserved sequences of the two monospores, and the completeness of the two strains was 99.66% and 98.97%, respectively (Table S3).

The whole genomes of the two monospores of *M. importuna* were annotated using the Nr(NCBI non-redundant database), InterPro, SwissProt, Gene Ontology (GO), and KEGG (Kyoto Encyclopedia of Genes and Genomes) databases, with the largest number of genes annotated in InterPro and Nr, more than 89% and 72% of the total, followed by the SwissProt function annotation and CDD (Conserved Domain Database) domain annotation. Finally, 10,497 and 10,947 genes, accounting for 91.13% and 93.01% of the total, were annotated as one defined function or domain (Table 2).

Species distribution of the Nr top hit showed the highest protein-number similarity between *M. importuna* and *T. melanosporum*. As morel and truffles belong to a different family, this result can be attributed to the unavailability of other genome data related to *Morchella* in the database [35]. The closest species to the proteins of *M. importuna* were *Parmelia omphalodes* and *Pseudogymnoascus pannorum*, with the former belonging to Pezizomycetes Pezizales Pyronemataceae, and the latter to the Pseudeurotiaceae family of Leotiomycetes, which clearly demonstrates the lack of studies of species more closely related to of *Morchella* (Table S4) [1,41].

Most enzymes were involved in the degradation of cellulose, hemicellulose, lignin, starch, and other biomasses belonging to the CAZyme family. In the whole genome of the two monospore strains, a total of 371 CAZYme genes were identified, including 174 and 173 glycoside hydrolases (GH), 70 and 69 glycosyltransferases (CT), as well as 22 and 23 polysaccharide lyases (PL), respectively. The protein numbers of carbohydrate lipases (CE) and carbohydrate domains (CMB) were equal in each category for the two monospore strains (29 and 46, respectively), and the number of auxiliary enzymes (AA) was 68 and 69, respectively. Compared with the number of CAZyme genes in the 15 reference strains, the total number of CAZYme genes in *M. importuna* was at a median level, and PL enzymes showed a significantly higher number in the strain of *M. importuna* than in the other strains, except for *Diplodia seriata* (Pezizomycotina Dothideomycetes) and *Pleurotus ostreatus* (Agaricomycotina Agaricomycetes). Morel was different from the ectomycorrhizal fungus, *T. melanosporum*, which lacks large sets of carbohydrate enzymes and contains only a few carbohydrate enzymes involved in the degradation of plant cell walls (Table S5).

Transcription factors (TFs) are essential components in regulatory networks. Thirty-seven TF families were identified in fungi, with six of them specific to the fungal kingdom. The number of putative transcription-factor genes (excluding general transcription factors regulating RNA polymerase) in filamentous fungi varies from 177 in *Phellodon confluens* to more than 600 to 800 in several *Fusarium* species [1,42]. No difference was found in the transcription factor type between the two strains. A total of 30 types of transcription factors were identified in the two strains, with 306 transcription factors identified in M04M24 and 297 in M04M26, which was obviously larger than the number of transcription factors identified in the adjacent species *T. melanosporum* (201) and *P. confluens* (177), and slightly less than that of *S. sclerotiorum* (330) and *Botrytis cinerea* (392–410). Similar to other filamentous ascomycetes, Zn2Cys6 was ranked first in the number of transcription factors, with 95 and 88 identified in the two monospore strains, followed by homeodomain-like, with an equal number of 44 in the two strains. For the four transcription factors of centromere, HMG, MADS-box, and helix-turn-helix, the M04M26 strain contained one more than the M04M24 strain. In contrast, the number of the seven transcription factors, Zn2Cys6, homeodomain-like, zinc, Myb, bZIP, GATA, and bromodomain was larger in the M04M24 strain than in the M04M26 strain (Table S6).

### 2.4. Genomic Structure and Characteristic Analysis

Transposable elements (TEs) are enigmatic genetic units with important roles in the evolution of eukaryotic genomes. They can mediate the genome evolution of nearly all species through mutation and chromosomal rearrangement or by modulating gene expression, and they account for approximately 3% of the yeast genome, about 58% of the truffle genome, up to 50% of the mammalian genome, and more than 80% of the genome of some plants such as wheat or maize [43]. The expansion of these elements is mediated by transposition events that can lead to their own duplication, and these transposition events are classified into two classes based on transposition mechanisms: RNA reverse transcriptional transposon (class I) and DNA transposon (class II). The total number of identified TEs in the genome of the two monospore strains of *M. importuna* was 1498 and 1979, with a total length of 2,175,089 bp and 3,951,540 bp, accounting for 4.25% and 7.73% of the genome size, respectively. Additionally, more repeat-like consensus sequences were found unable to be reliably classified, which occupied 7.36% and 7.65% of the M04M24 and M04M26 assemblies, respectively. These elements are referred to as “unknown” hereafter (Table S7) and were not used in downstream analysis. Nine DNA repeat elements were detected in the two monospore strains, namely CMC-EnSpm (12/0, the TE numbers of M04M24 and M04M26), PIF-Harbinger (34/3), TcMar-Pogo (1/0), TcMar-Tc1 (121/117), TcMar-Tc2 (0/12), TcMar-Tigger (0/45), hAT-Ac (156/246), hAT-Charlie (0/47), and Helitrons (16/2), one novel group of DNA transposons widespread throughout eukaryotes. The *M. importuna* repetitive element landscape was clearly dominated by class I transposons, which accounted for 77.3% of the total TEs in M04M24 and 76.1% in M04M26. The two monospore isolates were similar in the type of RNA retrotransposon elements, three long interspersed nuclear element (LINE), including I-Jockey (23/25), L1 (22/23), and Tad1 (387/502), and three long terminal repeat (LTR) families, including Copia (131/200), Gypsy (490/652) and Pao (85/82), and two short interspersed repeated sequence (SINE) orders, including transfer RNA (tRNA) (0/2) and tRNA-L2 (20/21) (Table S7).

Alternative splicing, a post-transcriptional regulation, can produce a good strengthening effect on the plasticity of the transcriptional group, the diversity of protein groups, and the complexity of gene expression, and there are a wide variety of alternative splicing events in eukaryotes [44]. With the help of the transcriptional sequence data, a total of 11,519 and 11,770 genes were predicted for the two monospore strains, with 703 (6.10%) and 670 (5.69%) of them being alternative splicing genes.

Most of the genes contained two alternative splicing sites, with 594 (42.2%) in M04M24 and 575 (43%) in M04M26, and the number of genes with more than two alternative splicing sites on a single gene was 108 (15.36% of the total alternative splicing gene) and 95 (14.18%) in the two strains, respectively (Table S8).

### 2.5. Genome Comparative Analysis of the Two Opposite-Polarity Monospores of M. Importuna

**Figure 1:**
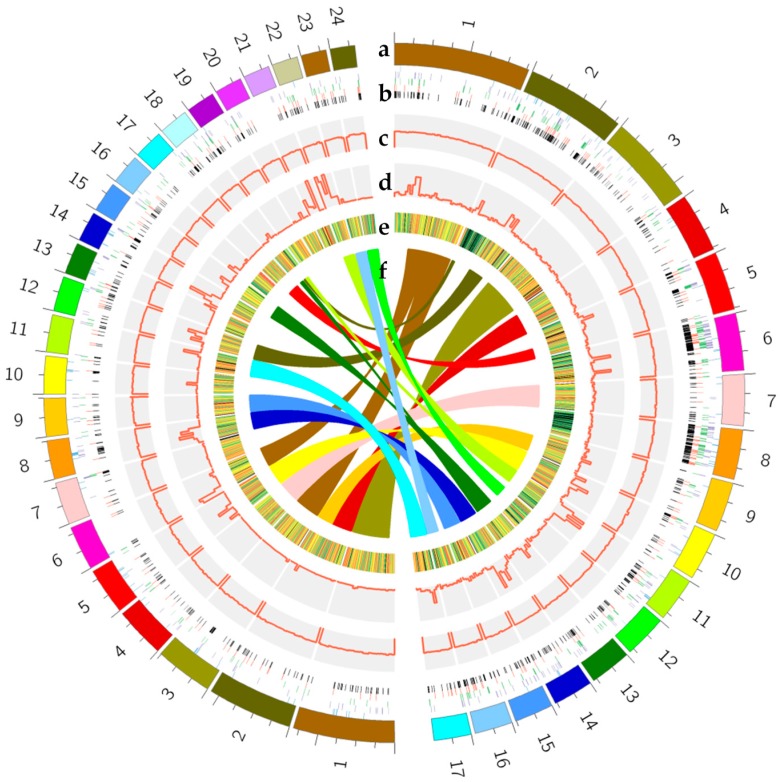
Genomic comparison and characteristic analysis of the two opposite-polarity monospore strains of *Morchella importuna*. Genomic comparison and characteristic analysis of the top N50 sequences between the two monospore strains, M04M24 and M04M26. The outermost loop is the scaffold fragment of the two strains, with different scaffolds indicated in different colors; M04M24 is on the right, M04M26 is on the left. The scaffolds that were larger than the respective N50 values are numbered 1 to 17 for M04M26, and from 1 to 24 for M04M24. The innermost circle (**f**) is the gene collinearity between the two strain scaffolds of the outermost ring (**a**), and the inner color corresponds to the outer color of the scaffold. (**b**) shows the distribution of the four different types of repeat sequences in the genome, and from outside to inside are the three RNA repeat sequences: long terminal repeat (LTR) green lines, long interspersed nuclear element (LINE) purple lines, short interspersed nuclear element (SINE) blue lines, and DNA repeat sequences: red lines, with black lines in the innermost cycle displayed as the synthesis of four repeat sequences. (**c**) (the histogram) represents the percentage of GC in the genome. (**d**) shows the single-nucleotide polymorphism (SNP) frequency of the genome in the form of a histogram, and the ordinate is the SNP number per 20 kb of genome. (**e**) shows the gene expression of RNA sequencing (RNA-seq) samples in the form of heat map. Red indicates that FPKM (Fragments Per Kilobase Million) is more than 100, orange is 100 > FPKM > 10, green is 10 > FPKM > 0, and black indicates FPKM = 0.

The two opposite-polarity monospore strains M04M24 and M04M26 were deep genome sequenced to facilitate the sequence and structural variation analysis. By comparing the M04M24 sequence reads to the M04M26 genome, 18,438 single-nucleotide polymorphism (SNP) mutation sites were detected in the M04M26 genome. The average mutation rate was 2752 bp per mutation site, with single-base mutations being 8969, the sequence length of deletion mutations being 1–117 bases, insertion mutations being 8020, and deletion mutations being 1562. These data can be conveniently applied to the development of SNP/indel markers (Figure 1).

A collinear analysis was conducted between the M04M24 and M04M26 genomes. The two monospore strains showed a good collinearity, with 99.22% of the sequences being well matched with each other (Table S9; Figure S1). When compared with the reference genome (the M04M26 genome), in the genome of M04M24, the number of one-to-one alignments was 943, the total length of the sequence was 49,745,378 bp, the average similarity was 99.87%, the number of M-to-M alignments was 3441, the total sequence length was 54,453,513 bp, and the average similarity was 99.23%; for feature estimates, the breakpoint, relocation, translocation, and inversion events were 5800, 16, 82, and 8, respectively. The insertion phenomenon of 1037 and 2565 loci occurred in the M04M24 and M04M26 strains, the total length of the sequence was 1,353,197 bp and 4,048,350 bp, and the average length of each insertion was 1304.92 bp and 1578.3 bp, respectively (Figure 1; Table S9).

**Figure 2:**
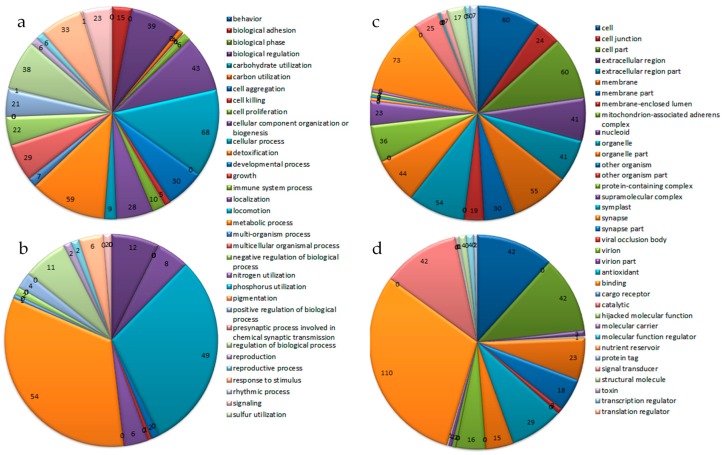
Gene ontology (GO) annotation of endemic genes in the two monospores of *Morchella importuna*. (**a**,**b**) Biological process classification; (**c**,**d**) Cellular component and molecular function classification; (**a**,**c**) represent the M04M24 strain; (**b**,**d**) represent the M04M26 strain.

The number and functional classification of endemic genes varied in the two monospore strains. In the 70 GO subcategories, the 667 endemic genes in the M04M24 strain were grouped into 47 subclasses, and the 868 endemic genes in the M04M26 strain were classified into 35 subclasses. In the M04M24 strain, the unique functional groups included nine biological process categories and four cellular component categories, and the endemic genes were mainly grouped under five subclasses. In the M04M26 endemic genes, only the anti-oxidant subclass was unique and it contained only one endemic gene. For the number of endemic genes, there were 26 more GO subclasses in the M04M24 strain than in the M04M26 strain, which were concentrated under the two large groups of cellular components and biological processes, such as the extracellular region part (the same GO number ratio of the two strains 41/1), extracellular region (41/3), cellular component organization or biogenesis (43/8), membrane (55/23), and organelle part (44/15). The M04M26 strain had more genes than the M04M24 strain in the two molecular function GO subclasses, including catalytic (25/42) and binding (73/110) (Figure 2).

As GO categories at a high level in the GO hierarchy were not very informative, the functions of the endemic genes in the two monospore strains were further analyzed separately by GO and KAAS enrichment. GO enrichment showed that the endemic genes of M04M24 and M04M26 were enriched in 362 and 9 GO categories (*p* < 0.01), respectively, and the top five enrichment categories in M04M24 included the following: extracellular region part (*p* = 1.37 × 10^−66^), extracellular organelle (*p* = 5.28 × 10^−54^), extracellular region (*p* = 2.48 × 10^−44^), cell junction (*p* = 2.51 × 10^−39^), and multicellular organismal process (*p* = 4.64 × 10^−38^). The top five enrichment categories in M04M26 were nucleic-acid binding (*p* = 2.08 × 10^−8^), heterocyclic-compound binding (*p* = 5.75 × 10^−7^), organic-cyclic-compound binding (*p* = 5.93 × 10^−7^), and DNA integration (*p* = 0.0001) and binding (*p* = 0.001) (Table 3). The KAAS pathway enrichment results showed that the endemic genes of M04M26 were not enriched, but those of M04M24 were enriched in 14 function categories at the level of *p* < 0.05, with the main enrichment categories being ECM-receptor interaction (*p* = 6.20 × 10^−8^), estrogen signaling pathway (*p* = 6.67 × 10^−6^), and focal adhesion (*p* = 8.94 × 10^−6^) (Table S10).

### 2.6. Phylogenetic Tree Construction and Evolutionary Divergence Time Analysis

**Figure 3:**
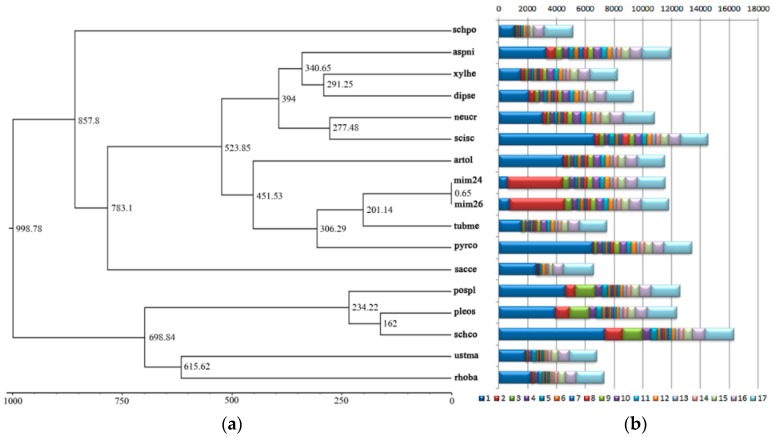
Evolutionary divergence time and statistical analysis of homologous genes in 17 species. (**a**) The maximum-likelihood method and LG + I + G + F model were used to construct the phylogenetic tree using 1072 single-copy genes identified from the two different monospore strains and 15 reference species. The abscissa unit was million years ago (Mya); the 15 species were schpo *Schizosaccharomyces pombe*, aspni *Aspergillus niger*, xylbe *Xylona heveae*, dipse *Diplodia seriata*, neucr *Neurospora crassa*, sclsc *Sclerotinia sclerotiorum*, artol *Arthrobotrys oligospora*, mlm24 *Morchella importuna* M04M24, mlm26 *Morchella importuna* M04M24, tubme *Tuber melanosporum*, pyrco *Pyronema confluens*, sacce *Saccharomyces cerevisiae*, pospl *Postia placenta,* pleos *Pleurotus ostreatus*, schco *Schizophyllum commune*, ustma *Ustilago maydis*, and rhoba *Rhodotorula graminis* (The reference genome used in this paper was downloaded from the Joint Genome Institute (JGI) website https://genome.jgi.doe.gov/programs/fungi/, Table S11). (**b**) The number of homologous genes of the corresponding 17 species is displayed by the stacking column pattern, and the number of homologous genes that appeared in the corresponding number of species is expressed in different colors.

A phylogenetic tree was constructed using the maximum-likelihood method and the LG + I + G + F model with 1072 single-copy genes identified in the orthology analysis. The tree topology was generally consistent with previous studies [37]. In the available sequenced genomes, *Tuber melanosporum* was the closest relative of the *Morchella importuna* species in one clade [35]. Based on fossil calibrations at the two calibrated nodes, including the ancestors of *Schizophyllum commune* and *Pleurotus ostreatus*, *Aspergillus niger*, and *Neurospora crassa*, the divergence time of *M. importuna* and *T. melanosporum* was estimated to be approximately 201.14 million years ago (Mya). The gene evolution and the single-copy homologous genes were different in the two monospore strains. The phylogenetic tree and divergence time analysis showed that the genetic distance of the two monospore strains was closest to one of the smallest branches, and the divergence time between them was about 0.65 Mya (Figure 3a).

The classification of homologous genes using the OrthoMCL software [45] within the 17 strains showed a clearly larger number of genes in morels than in the other strains, with 4418 and 4560 in the two monospore strains M04M24 and M04M26, the number of which was less than in *Schizophyllum commune* (7360), *Sclerotinia sclerotiorum* (6623), and *Pyronema confluens* (6522), and similar to that in *Postia placenta* (4603) and *Arthrobotrys oligospora* (4473). All homologous genes were clustered in one clade, excluding the mycorrhizal fungi *Tuber melanosporum* (1552), which had relatively few specific genes. The number of common genes of the 17 strains was approximately equal, with an average of 1961, and the two monospore strains had 1916 and 1908 common genes, respectively (Figure 3b).

The clustering analysis of gene contraction and expansion of the 17 strains showed that, when compared with *T. melanosporum*, a total of 30 clusters of orthologous genes (COGs) had obvious contraction and expansion in *M. importuna* (with the two monospore strains considered as a whole) at the *p* < 0.01 level. Specifically, 28 COGs expanded, such as COG30 (Nr: retrotransposon nucleocapsid protein, 7/0, *p* = 0), COG54 (Nr: retrotransposon nucleocapsid protein, 13/0, *p* = 0), COG4285 (Nr: hypothetical protein, 5/0, *p* = 0), COG17 (Nr: hypothetical protein, 4/1, *p* = 0.000005), and COG5 (Nr: Ankyrin repeat-containing domain protein, 19/0, *p* = 0.001207), and 28 COGs contracted, including COG176 (Nr: carbohydrate-binding module family 19 protein, 0/2, *p* = 0.00219), and COG2651 (Nr: alpha/beta-hydrolase, 0/2, *p* = 0.00219).

The two monospore strains also contracted and expanded at the whole-gene level. The M04M24 strain had an obvious contraction and expansion of nine COGs and 14 COGs, respectively (*p* < 0.01), with an obvious contraction for COG5 (Nr: Ankyrin repeat-containing domain protein, 22/19, *p* = 0), COG337 (Nr: Actin, 3/1, *p* = 0), and COG468 (Nr: ATP-dependent molecular chaperone HSC82, 3/1, *p* = 0), and an obvious expansion for COG17 (Nr: hypothetical protein, 0/4, *p* = 0). In the M04M26 strain, a total of five COGs contracted and 19 COGs expanded, with an obvious expansion for COG172 (Nr: telomere-associated RecQ helicase, 5/3, *p* = 0), COG4288 (Nr: retrotransposon nucleocapsid protein, 8/3, *p* = 0), COG5020 (Nr: transposase family Tnp2 protein, partial, 5/3, *p* = 0) and COG5021 (Nr: transposase family Tnp2 protein, partial, 6/3, *p* = 0). It is noteworthy that COG17 expanded in M04M26 (65/4, *p* = 0), but obviously contracted in M04M24 (0/4, *p* = 0), suggesting its unique role in the monospore strain M04M26. The MIM04M26Gene02976 (COG17) protein sequence was used to BLASTP in the NCBI database, and the possible function of the protein was identified as glutamate decarboxylase 2 (*Trametes pubescens*; E-value = 1 × 10^−33^, OJT07805.1), accompanied by Zn-finger domain-containing. We speculate that COG17 may play a special role in different monospore strains of *M. importuna* (Table S12).

## 3. Discussion

### 3.1. De Novo Genomic Sequencing Assembly and Annotation

The *Morchella* sp. belongs to the Ascomycota, Pezizomycetes, Pezizales, Morchellaceae, and *Morchella* genus [33,37]. To our best knowledge, the detailed genome data of Morchellaceae are yet to be reported. Apart from the release of some framework maps in public databases, such as the Joint Genome Institute (JGI) 1000 Genome Project concerning the two sets of *Morchella* (*Morchella conia* and *Morchella importuna*) and one *Gyromitra esculenta* strain, no in-depth analysis was carried out into the genome data. Chai et al. (2017) studied and published the mating type of *M. importuna*, Mel-15, and Mel-21 using genomic sequencing, but failed to provide detailed genomic information [27]. For Pezizales, 15 framework maps were released in JGI public databases (update 20180403, https://genome.jgi.doe.gov/mycocosm/species-tree/tree;hMUvDc?organism=fungi), and the detailed genomic information of *Pyronema omphalodes and Tuber melanosporum,* belonging *to* Pezizaceae and Tuberaceae, respectively, were published and analyzed in depth [1] [35] (Figure S2 and Table S11). Recent cytological and molecular evidence suggests that the ascospore of *M. importuna* is homokaryon, with each containing one mating-type gene MAT1-1 or MAT1-2 [4,27]. In order to obtain the complete genome information of *M. importuna*, two single spores M04M24 and M04M26 with a different mating type were separated from a mature ascocarp for deep genome sequencing analysis. In this paper, the de novo sequencing, fine physical mapping, and functional annotation were performed for the first time for the two opposite-polarity monospore strains of the commercially cultivated strain *M. importuna* M04.

The Kmer distribution of the two monospore strains was analyzed according to GCE’s and ALLPATHS-LG’s genome size assessment program. The results showed that the genome size was estimated to be 54.15 Mb, with repeat sequences covering 27.30% of the whole genome of *M. importuna*. In this paper, under de novo assembly, the actual assemblies were 48.98 Mb and 51.07 Mb for the two monospore strains, respectively, accounting for 90.45% and 94.31% of the whole genomes (Table 1). The BUSCO software was used to predict the completeness of gene content based on 290 conserved genes in the fungal database [46]. The two monospore strains were predicted to contain 284 and 283 complete BUSCO genes, four and five fragmented BUSCO genes, with three and one BUSCO genes missing, respectively, and the completeness of the genes was 99% (287/290) and 99.7% (289/290) (Table S3). About 5% of the genome was not completely assembled, probably due to highly repeated ribosomal RNA (rRNA) sequences, telomere structure, centromere structure, and a higher copy number of mitochondrial sequences in *M. importuna*. Through the coverage and gene prediction of sequences, the length of rRNA sequence was deduced to be about 561 kb on the scaffold Morimp01GS073, with a median base coverage of 2234×.

Sequence clustering analysis was performed using 500-bp sequence at both ends of each scaffolds of the two monospore strains with Clustalx [47] and Mega4.0 [48] software, combined with the conserved structural features of the eukaryotic telomere repeat sequence (TTAGGG/CCCTAA), which is also found in all vertebrates [49]. We found 16 (TTAGGG)n repeat sequence domains at one end of the scaffold (Figure S3), and nine (CCCTAA)n repeat sequence domains at the other end of the scaffold of the M04M26 genome (Figure S4), where the scaffold_Morimp01GS018 double end had (TTAGGG or CCCTAA)n repetition, indicating that it was a complete chromosome sequence. The length of repeat sequence was from 112 bp to 436 bp. In the M04M24 genome, only three different (TTAGGG or CCCTAA)n repeat sequence domains were found. This may be mainly due to the insufficient effect of the second-generation sequencing on long repeat sequences, and with the help of the third-generation sequencing protocol, more repetitive sequences can be effectively assembled for the M04M26 strain. From the repeated characteristics of telomere structure, we speculate that the chromosome number of *M. importuna* could be more than 16.

### 3.2. Mating-Type Locus of the Two Opposite-Polarity Monospore Strains of M. Importuna

In filamentous ascomycetes, the master regulators of sexual reproduction are the various genes that reside at the mating-type (MAT) loci and encode transcription factors that regulate the sexual cycle [50]. Heterothallic ascomycetes have a bipolar mating-type system, with isolates possessing one of the two non-allelic versions (idiomorphs) of a single MAT locus, termed MAT1-1 and MAT1-2. Conversely, homothallic ascomycetes carry both MAT loci within a single genome, with the two loci either fused together, located within close proximity, or on separate chromosomes [51]. The MAT1-1-1 and MAT1-2-1 genes encode transcription factors with a conserved alpha domain and high-mobility group (HGM) domain, respectively [50]. The genes *APN2*, encoding a putative DNA lyase, and *SLA2*, encoding a cytoskeleton assembly control factor, were reported to be adjacent to MAT loci in many filamentous ascomycetes [51].

**Figure 4:**
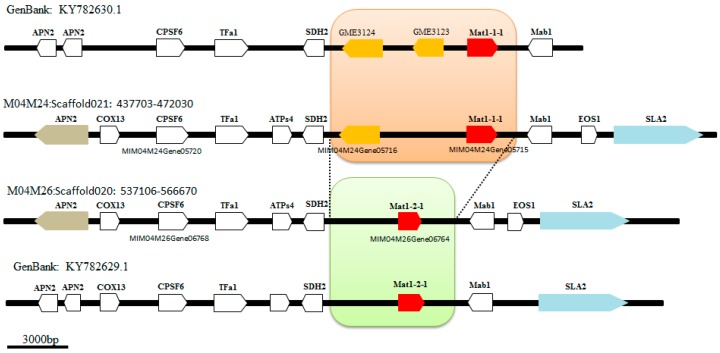
Structural analysis of two opposite-polarity monospore strains mating-type genes in *Morchella importuna*. The fine structure sketch was drawn manually based on the annotation information. M04M24 and M04M26 represent the mating loci of the two monospore strains used in this study. The sequences KY782629.1 and KY782630.1 reported in Chai et al. (2017) were downloaded from the National Center for Biotechnology Information (NCBI). The red box shows mating-type genes. The orange box represents the hypothetical gene. The flanking conserved genes are represented by *SLA2* with a light-gray box and the *APN2* gene with a pale blue purple. The sequence between the two dashed lines represents the largest different region of the two mating-type genes, with a sequence length of 6.6–11.9 kb. The ruler is 3000 bp.

Based on the previous verification results of mating type [4], the two monospore strains were sequenced to complete the mating type and flanking sequences, with the M04M24 strain containing a complete MAT1-1 locus (MI_M04M24_Scaffold021: 437703-471030) and the M04M26 strain containing a complete MAT1-2 locus (Morimp01GS020: 537106-565670). The flanking sequences of mating-type loci showed good collinearity, with high identity upstream (6252/6305 base, 99%) and downstream (15,227/15,293 base, 99%). There were differences for the MAT loci 6.6–11.9 kb in length between the two mating-type sequences (Figure 4). Two genes were predicted for the MAT1-1 mating-type loci, MIM04M24Gene05715 and MIM04M24Gene05716. NCBI BLASTP showed that the MIM04M24Gene05715 gene encoded 525 amino acids, contained MATalpha_HMGbox conserved domain (Interval:143–234, pfam04769, E-value = 8.38 × 10^−5^) and had 99% similarity with the APF29255.1 protein (mating-type MAT1-1 locus protein (*Morchella importuna*)) [27]. NCBI BLASTP also indicated that MIM04M24Gene05716 encoded 512 amino acids, and had 100% similarity with the AVI60818.1 hypothetical protein, but the specific function needs further study. Only one gene was predicted for the MAT1-2 mating-type loci of the M04M26 strain, MIM04M26Gene06764, which encoded 307 amino acids, had 100% similarity with the APF29258.1 protein (mating type MAT1-2 locus protein (*Morchella importuna*)) and contained MATA_HMG-box superfamily conserved domain (Interval:135–210 aa, cd01389, E-value = 2.74 × 10^−30^) as indicated by NCBI BLASTP [27]. Both flanking sequences of the mating-type loci were completely conserved, and the gene prediction and annotation displayed APN2-COX13-CPSF6-TFa1-ATPs4-SDH2- and -Mab1-EOS1-SLA2, respectively (Figure 4).

There were four main differences in the prediction of coding genes in *M. importuna* mating-type loci between this study and the study by Chai, et al. (2017) [27]. Firstly, in Chai’s report of MAT1-1 and MAT1-2 loci, two adjacent APN2-encoding proteins were predicted in the flanking sequence of each MAT locus, with an amino acid length of 286 and 320 aa, respectively. In this study, an independent APN2-encoding protein was predicted, with a length of 627 aa, and the BLASTP comparison showed that the two APN2 sequences reported by Chai can be compared to the two ends of the APN2 sequence in our study, with a similarity of 100%, *E*-value = 0 (Figure S5). *APN2* is a relatively conserved gene on one side of the MAT loci and the length varies slightly in different species, such as *Xylona heveae* 653 aa (XP_018186049) [52], *Phialophora attae* 601 aa (XP_018003209) [53], *Aspergillus bombycis* 627 aa (XP_022389684.1) [54], and *Cordyceps confragosa* 637 aa (OAA74996) [55]. Secondly, unlike the gene prediction in this paper, Chai’s report showed that there was no COX13 and ATPs4 protein in the flanking sequence of the MAT1-1 locus, while the MAT1-2 locus contained these two predictive proteins. Thirdly, despite the similarity between the two studies in prediction of location, domain, and annotation information of the *MAT1-1-1* gene, there were some differences in the MAT1-1 locus between the two studies. Specifically, in Chai’s report, the MAT1-1 mating-type loci had three genes: *MAT1-1-1*, *GME3124*, and *GME3123*, with the latter two presumed to be new mating genes of *M. importuna*; however, in the present study, a hypothetical gene MIM04M24Gene05716 was obtained only in this region, which was 100% similar to *GME3124* (AVI60818.1), but no conserved domain can be used to prove its function; thus, whether it is a mating-type gene remains to be verified. Finally, in Chai’s report, a *MAT1-2-1* gene was predicted in the MAT1-2 locus, with a length of 328 aa, while, in this paper, the amino-acid sequence alignment showed that the predicted gene had a length of 307 aa, sharing 100% similarity with Chai’s reported *MAT1-2-1* gene, but was 22 aa shorter at the C-terminal, while both also contained MATA_HMG-box conserved domain (Figure 4). The results of gene prediction varied greatly probably due to different prediction methods and parameters [27]. Our prediction was based on the sequence alignment of the transcriptional information and ab initio prediction [38]. The low expression of the genes in the mating-type loci may be the main cause for the deviation of genetic prediction in this area, while the limited known adjacent reference genome sequence may also be responsible for the major difference in the prediction of the mating type and the flanking sequence of *M. importuna*. The origin of the exact coding sites for mating type and flanking sequences needs to be further elucidated.

### 3.3. C.omparative Genome Analysis

While more than ten genome sequences were sequenced and are available for each class in Pezizomycotina (Sordariomycetes, Leotiomycetes, Eurotiomycetes, and Dothideomycetes), genome sequencing was only performed for one Orbiliomycetes and two Pezizomycetes species, namely the nematode-trapping fungus, *A. oligospora*, and the black truffle, *T. melanosporum*, and the saprobic Pezizomycete with a typical apothecium as the fruiting body [1,35,56]. *A. oligospora* (teleomorph *Orbilia auricolor*) belongs to a group of nematode-trapping soil fungi with a size of 40 Mb and encodes ~11,500 protein-coding genes, similar to the size and coding capacity of other ascomycete genomes [56]. Despite a larger size than the genome of the other sequenced ascomycetes, the *T. melanosporum* genome (125 Mb) is mainly occupied (~58%) by transposable elements, and only encodes ~7500 protein-coding genes in very rare multigene families [35]. With a size of 50 Mb and ~13,400 protein-coding genes, the *P. confluens* genome is more characteristic of higher filamentous ascomycetes than the truffle genome [1], which is closely related to *Morchella* in the evolutionary relationship under the same order Pezizales. The ~50-Mb genome of *M. importuna* encodes 11,519 (M04M24) and 11,770 (M04M26) protein-coding genes, similar to the size and coding capacity of the other ascomycete genomes.

Evolutionary trees based on a single or a few genes may create inconsistent topology, while those based on the series of available genes in the whole genome can provide relatively high resolution. In this study, we used 1072 genome-wide single-copy orthologous protein sequences combined with 15 reference strains to construct the maximum-likelihood tree at the higher level of *Morchella importuna* (Figure 3). The evolutionary tree showed that *M. importuna* and *T. melanosporum* were clustered to the smallest group, but the collinearity analysis by MCScanX showed a low collinearity between them; 279 collinearity blocks consisting of only 23.7% genes of *M. importuna* were identified with the threshold of at least five genes in one collinerity block, and the largest block only contained 39 genes. There was also a very low collinearity between *P. confluens* and *T. melanosporum*, with only about 11% of the genome sequence of *P. confluens* showing a syntenic sequence through alignment with the PROmer algorithm of MUMmer at the amino-acid level [1]. The low degree of synteny might be attributed to their large evolutionary distance, as well as different ecological types.

Based on ribosomal subunits (18S and 28S) and spacer sequences (ITS), the higher-order classification of *Morchella* was further studied in detail, and seven genera were included in the Morchaceae family [33]. In recent years, the genus *Morchella* was extensively studied with the development of the multigene molecular phylogenetic system [31,32,33]. The genus *Morchella* can be divided into three major branches, including an early diverging basal lineage Rufobrunnea clade, the Elata clade, and the Esculenta clade. *M. importuna* belongs to the Elata clade, with phylogenetic species formally named as Mel-10 [30].

Based on the phylogenetic analysis and diversification time study of the 45 representative strains with the conserved gene fragments (*RPB1*, *RPB2*, *EF-1a*, *LSU*, *28S rDNA*), the diversification time of the family Morchaceae was in the middle Triassic (243.63 Mya), and the genera *Morchella* and *Verpa* and *Disciotis* were differentiated in the early Cretaceous (129.61 Mya), with a basal monotypic lineage represented by *M. rufobrunnea* and two sister clades consisting of Elata Clade and Esculenta Clade. The early divergence of the remaining in-group taxa comprising the Elata and Esculenta Clades was dated at 101.78 Mya [32]. The ITS sequence and more specimens were added for further study of the phylogenetic and diversification time of the genus *Morchella* on the basis of the prophase multigene polygenic system, resulting in the discovery of a late Jurassic origin of the *M. rufobrunnea* lineage (154.15 Mya) and an early-to-mid-Cretaceous origin of the Esculenta and Elata Clades (123.46 Mya) mainly in western North America [31].

The lack of available fossil information and the evolutionary pressures of different genes in different species may be the cause for a possible deviation in the molecular clock model based on a small number of sequences. Genomic information enables us to make a more accurate analysis of the evolution and diversification time of species. Based on the 15 published species close to morel and the whole-genome sequence of *M. importuna* in this paper, a total of 1072 single-copy conserved genes were used to construct a species evolution tree and analyze the divergence time among species. The phylogenetic tree of the *Morchella* species is consistent with that of earlier studies [37]. *M. importuna*, *T. melanosporum*, and *P. confluens* are clustered under one branch, which belongs to Pezizales. It is generally believed that the most accurate time for determining species differentiation is based on the information from fossil specimens. The time of differentiation of the whole-genome single-copy gene shows that the divergence time of the morel and truffles was 201.14 Mya, which was slightly different from the report by O’Donnell in that they pushed the age of these lineages back in time about 30 million years [32]. Additionally, the divergence time of truffles with *P. confluens* was 306.29 Mya in this study, which is within the scope of previous studies [1]. The two single-spore strains represent two different sets of genes in *M. importuna*, and there was also a genetic differentiation between them, with a divergence time of about 0.65 Mya (Figure 3a,b).

### 3.4. The Two Monospore-Specific Genes Reveal the Possible Heterothallic Characteristics of M. Importuna

The arbuscular endomycorrhizal (AM) fungi *Rhizophagus irregularis* has a multinuclear cell structure, and its single-nucleotide sequencing results showed that the similarity between the two different nuclei was as high as 99.97%, with few SNPs and indels among the haploid nuclei [57]. Different nuclei of basidiomycetes of *Agaricus bisporus* showed different patterns in gene expression [58]. The sequence structure and gene structure varied in the different polar nuclei of *M. importuna*. Functional classification and enrichment analysis showed that the endemic genes of the two monospore monokaryon strains perform distinct functions. The function of M04M24-specific genes is mainly related to GO functional cellular component and biological process categories, including region part, extracellular region, cellular component organization or biogenesis, membrane, and organelle part. The endemic genes of M04M26 are mainly involved in the molecular function GO category of catalytic and binding. It seems, to some extent, that the endemic genes of M04M24 play a regulatory role in the outer layer of the cell, and the unique genes of M04M26 function directly in cells. Such assumptions are also supported by the enrichment of GO and KAAS functions (Table 3).

There is only one type of nucleus in each ascospore of *M. importuna*, and the mating-type structure information also shows that only one type of mating-type gene was contained in *M. importuna*, implying that *M. importuna* is a heterothallic fungus. However, there is still a lack of cross-matching data between the strains and direct cultural and fruiting evidence. The two monospore strains M04M24 and M04M26 have about 0.8% difference (collinearity 99.2%) at the genome level, and their specific genes are 5.79% and 7.37% of the total number of genes at the gene level, respectively. The evolutionary analysis also showed that the two nuclei have a genetic differentiation distance of 0.65 Mya at the molecular evolution level. The results showed that the complete life history of *M. importuna* should be that of a heterogeneous fungus, which is necessary for the nucleus to match with that of the other polarity. *T. melanosporum* is similar to *M. importuna*, and it is a kind of heterothallic fungus. Molecular biology studies showed that a mating-type mycelium of the tuber may play the role of the mother, while the opposite mating type mycelium performs the paternal role [59]. When investigating the mating-type genes in different parts of *Morchella*, only one mating-type gene was detected in the stalk tissue at any one time [26], suggesting that the sac fungi morel and the basidiomycetes may be distinctly different in their development process, and the mycelium fusion, the plasmogamy, and the karyogamy may be limited to a specific time and space. The complex relationship between the two different polar nuclei in the life cycle needs to be further studied.

## 4. Materials and Methods

### 4.1. Strain Selection and Material Preparation

*Morchella importuna* M04, a commercial strain, was identified by analysis of morphological characteristics and multigenic phylogenetic tree construction [31,33]. The strain was deposited in the Preservation Center of the Institute of Applied Mycology, Huazhong Agricultural University (Wuhan, China). A micromanipulator was used to pick up the monospore population from one ascocarp. The single monospore was cultured on a 9-cm petri dish containing a solid medium of complete yeast extract medium (CYM; glucose 20 g·L^−1^, yeast extracts 2 g·L^−1^, peptone 2 g·L^−1^, K_2_HPO_4_ 1 g·L^−1^, MgSO_4_ 0.5 g·L^−1^, KH_2_PO_4_ 0.46 g·L^−1^, and agar 20 g·L^−1^) to obtain the pure strain for further experiment, and a total of 58 single-spore populations were obtained. The polarity of the two monospore strains used in this experiment was verified by the mating type gene (identification of the mating-type gene was supported by the early genomic framework data of our laboratory), with the mating type of MAT1-2 for the monospore M04M24 and the opposite mating type of MAT1-1 for M04M26. The parental heterokaryon strain M04 was used for transcriptome sequencing to annotate the genes of the genome. The information of the strains was deposited in the NCBI (https://www.ncbi.nlm.nih.gov/) with BioSample numbers SAMN09514283 (M04M24), SAMN09514284 (M04M26), and SAMN09514285 (M04).

The monospore strains M04M24 and M04M26 were activated on the CYM solid medium under the laboratory conditions and transferred to the CYM liquid medium for static culture at 23 °C for seven days to obtain the mycelia. The parent strain M04 was used for transcriptional sequencing. Under the same culture conditions as described above, the mycelia initially developed with vegetative mycelia after incubation at 23 °C for three days, followed by the emergence of initial sclerotia on the surface of the medium at seven days of incubation, and development into mature sclerotia at 14 days of culture. Finally, a total of three samples were pooled together for RNA sequencing (RNA-Seq) in order to obtain a higher number of genes. The cultured mycelia were rinsed with clean pre-cooled distilled water, dried with clean dry filter paper, then immediately frozen in liquid nitrogen, and stored at −80 °C for the subsequent experiments.

### 4.2. Genome and Transcriptome Sequencing

The two monospore strains were sequenced using different schemes. The monospore M04M26 was sequenced using the Illumina Hiseq 4000 sequencing platform as follows: 3 Gb raw data were generated from two insert fragments of 270 bp in length, PCR-free library, and sequence type PE150; 3 Gb raw data were produced from one 10-kb mate-pair library and sequencing type PE50. Meanwhile, the PacBio RSII sequencing platform (PacBio P6-C4) was used for third-generation sequencing of M04M26, and the raw data of two cells and 5.3 Gb of subread data from two SMRT cells were generated from the length of the 20-kb library (Table S1).

The M04M24 sequencing scheme was slightly different from that of M04M26, with the 270 bp and 800 bp libraries consisting of two small fragments, and the 6-kb library composed of a large fragment, and the sequence types of the three libraries were PE150, PE125, and PE50 under the Illumina Hiseq 4000 sequencing platform, respectively. The raw data were all 4 Gb. In order to facilitate the prediction of the genes across the whole genome, the transcriptional sequencing of the parent heterokaryon strain M04 was carried out. The sequencing scheme used was the Illumina Hiseq 4000 sequencing platform with the insert fragment length 270 bp and the PE150 sequencing type, resulting in the generation of 6 Gb raw data. All reads were deposited in the Sequence Read Archive (SRA) at NCBI, under accession number SRR7448150-SRR7448158.

### 4.3. Genome Assembly and Annotation

Using high-throughput sequencing data, the genomic sequences of M04M24 and M04M26 were de novo sequenced and assembled. Because of the use of both the Illumina and PacBio platforms for sequencing the monospore M04M26, a special genome assembly scheme was adopted. The de novo genomic assembly was performed in three independent steps. Firstly, the software Trimmomatic (version: trimmomatic-0.33) was used to combine and filter the raw reads [60], followed by the use of the ALLPATHS-LG software (version: 44849) for the de novo genomic assembly with the Illumina data [61], the DBG2OLC software to mix the Illumina and PacBio data for the de novo genomic assembly [62], and the FALCON software for the de novo genomic assembly with the PacBio data only. Next, Quickmerge software was used to integrate the results of the above three genome assemblies [63]. Finally, SSPACE software (version: SSPACE_Standard_v3.0) was used to connect scaffold with Illumina mate-pair data [64], and GAPCLOSER software (version: BGI, v1.12) was used to fill gaps in the assembly with the Illumina paired-end data [65], followed by the use the finisherSC software to connect the scaffolds and fill the gaps with the PacBio data [66]. The final M04M26 genome was obtained using Pilon (version: pilon-1.22) and quiver software for SNP and indel error correction [67]. For M04M24, only Illumina sequencing was carried out, and only ALLPATHS-LG software was used for the de novo genomic assembly (detailed genome analysis flow and parameter settings can be found in Supplementary Materials 1 and 2).

The same strategy was adopted for structural and functional annotation of the genomic sequence of the two different monospore strains M04M24 and M04M26. Firstly, based on the sequence similarity principle, the repeat sequence of the genome was identified by comparing the genome sequence with the repeat sequence of the Repbase database using the RepeatMasker software [68,69]. Meanwhile, the RepeatModeler software was used to compare the genome sequence with itself to predict the repetitive sequences with a repetition of at least 15 times in the genome sequence (http://www.repeatmasker.org/RepeatModeler/). Then, the repetitive sequence loci predicted in the genome by the two above software were soft-masked (as the masked sequences were converted to lowercase letters) for subsequent data analysis, followed by the use of the Hisat2 software to set the “--min-intronlen 20--max-intronlen 4000--score-min L, 0, -0.4” parameters to compare the sequence of Illumina reads of the transcriptional data with the genome [70]. The comparison results of the SAM format and the sequence of the genome were input into the BRAKER2 software, and the parameter “--fungus” was set up for predicting the structure of the protein-encoding genes [38]. Then, based on the similarity of the protein sequence, the amino-acid sequences of the predicted proteins in the whole genome were compared with the Nr, SwissProt, KOG, and KEGG database using the BLAST method for gene annotation. Next, based on the similarity of protein domains, the genome CDD and InterPro annotation of the predicted proteins were carried out using the HMM method. Finally, based on Nr and InterPro annotations, the Gene Ontology (GO) annotation of the genes was performed with the Blast2go software [71].

Fungi are used as decomposers in the ecological environment, with CAZyme enzymes playing an important role in the degradation process. Therefore, the members of the CAZyme family across whole genome were specially analyzed in this paper. Through the dbCAN V6.0 software, all the protein sequences of the CAZyme family were collected using the CAZy database (http://www.cazy.org) and the HMM domain information of the entire CAZyme family was obtained using the HMM algorithm. Based on the two dbCAN databases, the protein sequence database was first compared using the BLASTP method, then the HMM database was compared with the Hmmscan method, and the results from the intersection of the two methods were considered as accurate [72]. On this basis, the threshold of the BLASTP and Hmmscan software for each gene family was further calculated to obtain the more credible results from the two independent methods.

The transcription factor family was annotated by the InterPro number. Additionally, the completeness of the genome and the reliability of the gene prediction were evaluated by analyzing the predicted protein sequence of the whole genome using the Benchmarking Universal Single-Copy Orthologs (BUSCO) software with a single-copy homologous gene database named fungi_odb9 [46].

### 4.4. Phylogenetic Tree Construction and Evolutionary Divergence Time Analysis

Comparative genomic analysis was carried out using the genome of the two monospores (M04M24 and Mm04M26) of *M. importuna* and 15 other previously published fungi. Firstly, the OrthoMCL v2.0.9 software was used to set the default parameters for the orthology and paralogy analyses, followed by the use of the MCL v12-068 software to cluster the homologous genes [45]. Based on the above result, all single-copy genes were extracted, followed by multiple-sequence alignment using the MAFFT v7.158b software [73], merging the results and extracting the conserved blocks with the default threshold set by the Gblocks v0.91b software [74]. Then, the Prottest v3.4 software was used to calculate the best amino-acid replacement matrix [75], and the RAxML version 8.1.24 software was used to calculate the maximum-likelihood species trees [76].

Dating analysis was performed using the r8s software [77], with one fossil calibrated point at 162 millions of years ago (Mya) between *Schizophyllum commune* (Agaricomycotina Agaricomycetes) and *Pleurotus ostreatus* (Agaricomycotina Agaricomycetes), and the other calibrated point at 394 Mya between *Aspergillus niger* (Pezizomycotina Eurotiomycetes) and *Neurospora crassa* (Pezizomycotina Sordariomycetes) [78,79]. After transforming the evolution species trees into a super tree, the divergence time of each node was obtained. Finally, the gene family expansion and contraction from OrthoMCL was analyzed using the Cafe software [45,80] (Supplementary Material 3).

### 4.5. Comparative Analysis of the Two Mating-Type Monospore Genomes

The genomic nucleic-acid sequences of M04M24 and M04M26 were compared and collinear-analyzed by MUMmer [81]. The protein sequences between the two strains were compared using the OrthoMCL v2.0.9 software to obtain the common genes and endemic genes [45]. The endemic genes were analyzed using MCScanX and visualized using Circos [82,83]. According to the results of genome annotation, the mating-type genes of the two monospore strains were manually analyzed.

The Trimmomatic v0.33 software was used to set the parameter “ILLUMINACLIP: /PATH/TruSeq3-PE.fa:2:30:10 LEADING:3 TRAILING:3 SLIDINGWINDOW:4:15 MINLEN:50 TOPHRED33” to control the paired-end data of the monospore M04M24 using the Illumina Hiseq 4000 platform [60]. Then, the HISAT2 v2.1.0 software was used to set the parameter “--score-min L, -0.3, -0.3” for comparison of the Illumina Hiseq 4000 sequencing data between M04M24 and M04M26 [70], and the HaplotypeCaller command in the GATK v4.0.3.0 software was used to set the parameter “-ERC NONE” to obtain the SNP/indel information in the M04M26 genome. Finally, the VariantFiltration command was used to set the following thresholds to filter SNP/indel: QD < 13; MQ < 20; FS > 20; MQRankSum < −3.0; ReadPosRankSum < −3.0 [84]. The same parameters and methods were used to compare the Illumina Hiseq 4000 sequencing data of strain M04M26 with the genomic sequence of M04M24 to obtain the SNP/indel information in the M04M24 genome (Supplementary Material 3).

## Figures and Tables

**Table 1:** De novo genomic assembly results of the two opposite-polarity monospores of *Morchella importuna*.

Description	M04M26	M04M24
Genome scaffold number (*n*)	106	394
Genome contig number (*n*)	111	763
Longest length (bp)	3,559,784	2,650,670
Shortest length (bp)	13,107	990
Genome scaffold size (bp)	51,078,152	48,978,911
Genome contig size (bp)	51,061,582	48,728,179
Rate of N	0.000324405	0.005119183
Rate of GC	0.473300338	0.472875746
Scaffold N50 (bp)	978,718	653,797
Contig N50 (bp)	951,626	198,406
Scaffold N90 (bp)	265,726	167,990
Contig N90 (bp)	261,029	47,755
Genome completeness (%)	94.31	90.45
Number of sequences ≥ 1 kb (*n*)	106	392
Number of sequences ≥ 2 kb (*n*)	106	252
Number of sequences ≥ 3 kb (*n*)	106	215

**Table 2:** Gene annotation results of the two opposite-polarity monospore of *Morchella importuna*.

Database	M04M24	Percentage (%)	M04M26	Percentage (%)
Nr	8357	72.55	8517	72.36
InterPro	10364	89.97	10,577	89.86
KOG	5990	52.00	6000	50.98
COG	5795	50.31	5816	49.41
SwissProt	6292	54.62	6300	53.53
GO	6173	53.59	6219	52.84
KAAS	3459	30.03	3436	29.19
CDD	7162	62.18	7154	60.78
Total	10,497	91.13	10,947	93.01
Whole genome	11,519	100.00	11,770	100.00

**Table 3:** The main GO enrichment results of endemic genes in the two opposite-polarity monospore of *Morchella importuna*. Pop—population; ID—identifier.

M04M24						
ID	Pop Total	Pop Term	Study Total	Study Term	*p*	Name
GO:0044421	6171	54	104	41	1.37 × 10^−66^	Extracellular region part
GO:0043230	6171	46	104	36	5.28 × 10^−54^	Extracellular organelle
GO:0005576	6171	133	104	41	2.48 × 10^−44^	Extracellular region
GO:0030054	6171	29	104	24	2.52 × 10^−39^	Cell junction
GO:0032501	6171	57	104	29	4.64 × 10^−38^	Multicellular organismal process
GO:0031982	6171	130	104	38	2.59 × 10^−35^	Vesicle
GO:0099080	6171	54	104	23	1.38 × 10^−27^	Supramolecular complex
GO:0032502	6171	151	104	30	5.10 × 10^−25^	Developmental process
GO:0005886	6171	135	104	27	3.68 × 10^−22^	Plasma membrane
GO:1903561	6171	46	104	36	1.44 × 10^−20^	Extracellular vesicle
GO:0071944	6171	211	104	31	1.56 × 10^−20^	Cell periphery
GO:0022610	6171	32	104	15	5.82 × 10^−19^	Biological adhesion
GO:0008219	6171	32	104	15	6.81 × 10^−18^	Cell death
GO:0034330	6171	15	104	13	2.97 × 10^−16^	Cell junction organization
GO:0009888	6171	24	104	22	6.76 × 10^−16^	Tissue development
GO:0009611	6171	16	104	14	7.52 × 10^−16^	Response to wounding
GO:0048869	6171	125	104	22	1.44 × 10^−15^	Cellular developmental process
GO:0032879	6171	60	104	17	4.61 × 10^−15^	Regulation of localization
GO:0040011	6171	11	104	9	5.78 × 10^−15^	Locomotion
GO:0048471	6171	13	104	10	4.70 × 10^−14^	Perinuclear region of cytoplasm
**M04M26**						
**ID**	**Pop Total**	**Pop Term**	**Study Total**	**Study Term**	***p***	**Name**
GO:0003676	6216	981	167	69	2.08 × 10^−8^	Nucleic-acid binding
GO:1901363	6216	1829	167	84	5.76 × 10^−7^	Heterocyclic-compound binding
GO:0097159	6216	1830	167	84	5.94 × 10^−7^	Organic-cyclic-compound binding
GO:0015074	6216	39	167	7	1.26 × 10^−4^	DNA integration
GO:0005488	6216	3400	167	110	0.00130	Binding
GO:0006259	6216	282	167	11	0.00661	DNA metabolic process
GO:0019843	6216	26	167	2	0.00770	Ribosomal RNA (rRNA) binding
GO:0010257	6216	1	167	1	0.00913	NADH dehydrogenase complex assembly
GO:0000256	6216	3	167	1	0.00977	Allantoin catabolic process

## Supplementary Materials

Supplementary materials can be found at http://www.mdpi.com/1422-0067/19/9/2525/s1.
