# Supplementary figures and images for: Opposite Polarity Monospore Genome De Novo Sequencing and Comparative Analysis Reveal the Possible Heterothallic Life Cycle of Morchella importuna
Source: Int J Mol Sci. 2018 Aug 25;19(9):2525. doi: 10.3390/ijms19092525 (PMC6164635; doi:10.3390/ijms19092525)

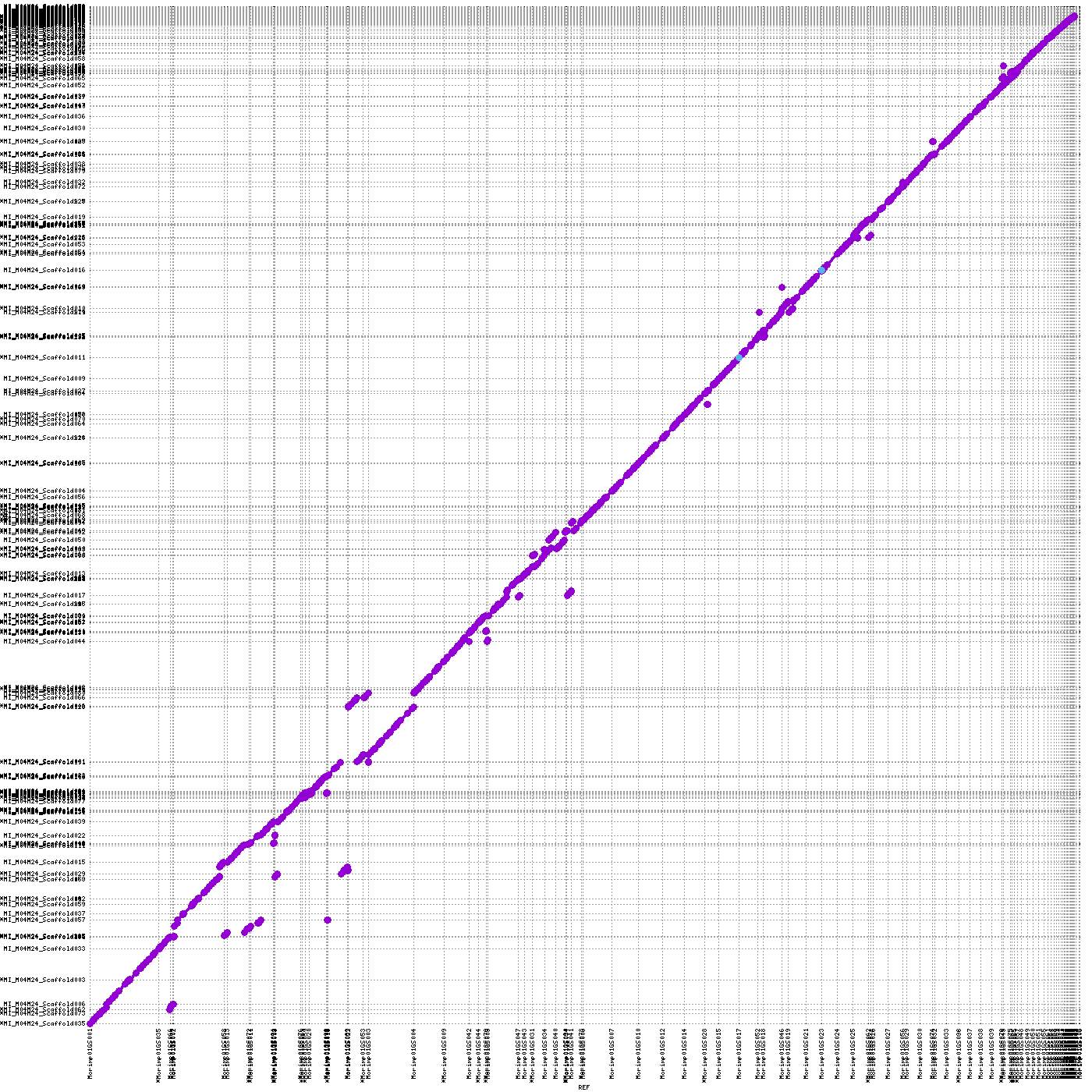

Supplement: Supplementary file 1 [file ijms-19-02525-s001.zip › Supplement/Figure S1 MIM04M24_vs_MIM04M26 colinearity.png]

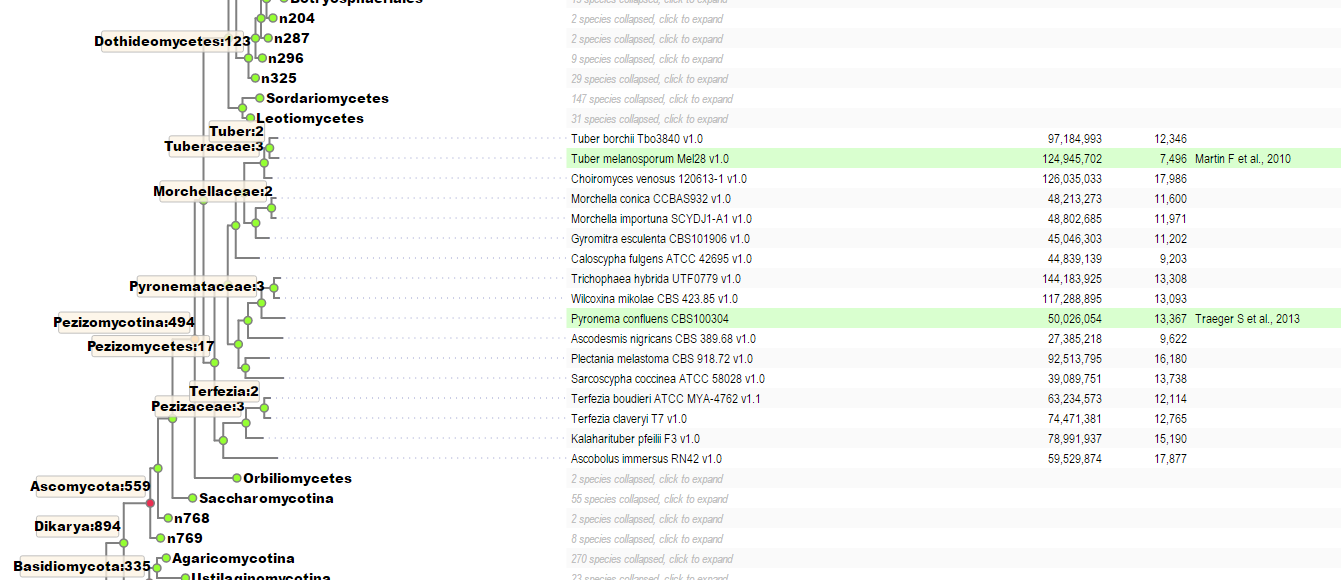

Supplement: Supplementary file 1 [file ijms-19-02525-s001.zip › Supplement/Figure S2 JGI publication genome project of Pezizomycetes.png]

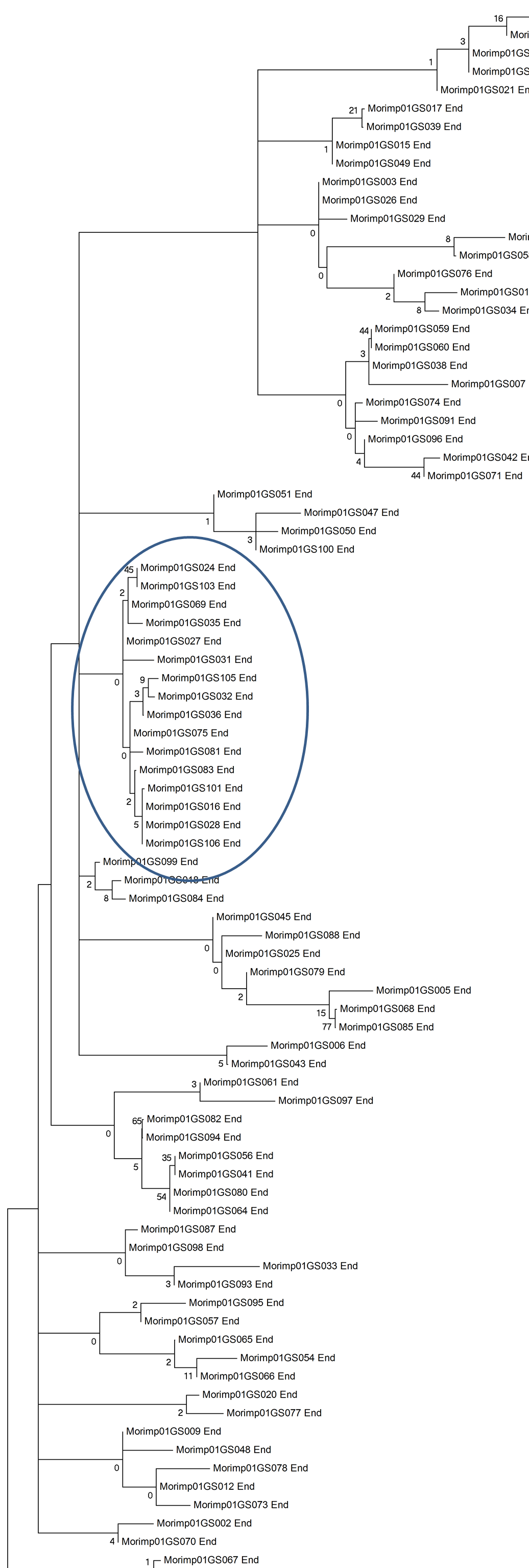

Supplement: Supplementary file 1 [file ijms-19-02525-s001.zip › Supplement/Figure S3 Cluster analysis of telomere structure 500end of scaffold.png]

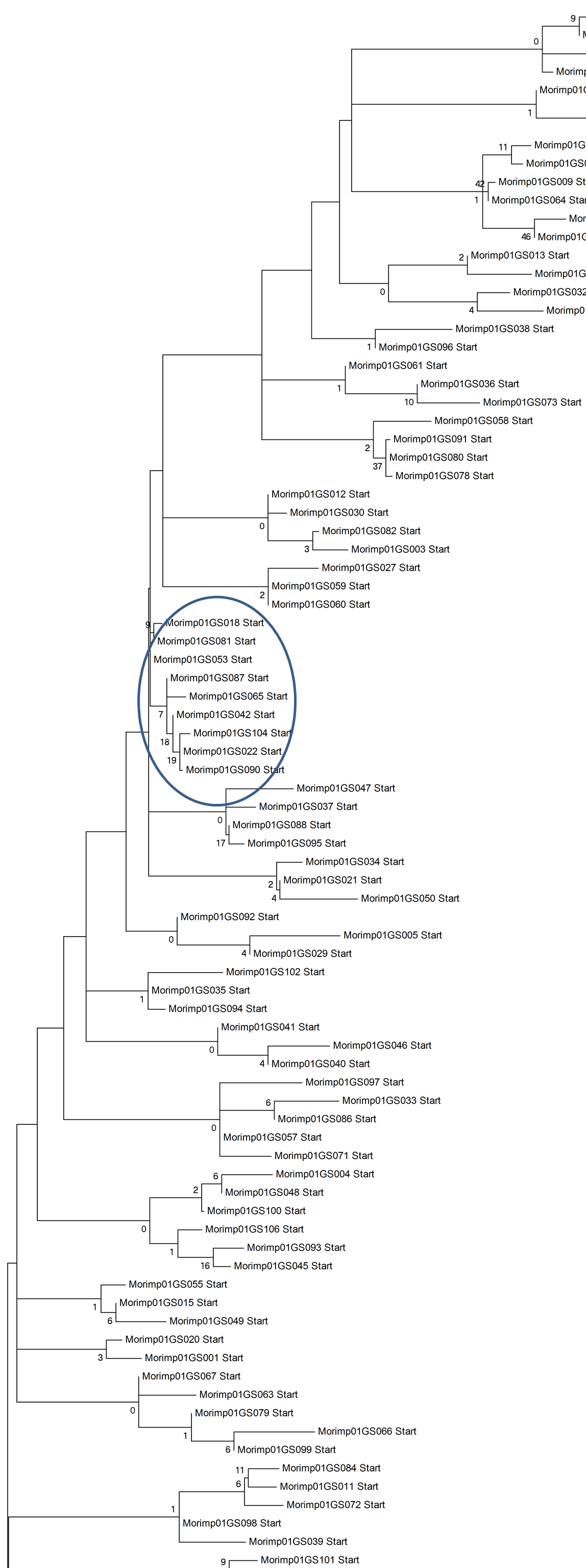

Supplement: Supplementary file 1 [file ijms-19-02525-s001.zip › Supplement/Figure S4 Cluster analysis of telomere structure 500start of scaffold.png]

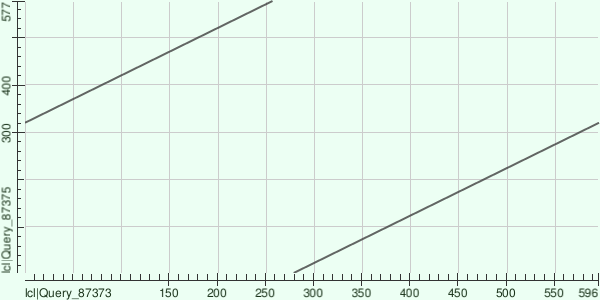

Supplement: Supplementary file 1 [file ijms-19-02525-s001.zip › Supplement/Figure S5 APN2 blastp result of our APN2 aa to Chens two APN2 sequences.png]
