# Supplementary material for: Opposite Polarity Monospore Genome De Novo Sequencing and Comparative Analysis Reveal the Possible Heterothallic Life Cycle of Morchella importuna
Source: Int J Mol Sci. 2018 Aug 25;19(9):2525. doi: 10.3390/ijms19092525 (PMC6164635; doi:10.3390/ijms19092525)
Supplement: Supplementary file 1 [file ijms-19-02525-s001.zip › Supplement/Supplementary Material 1.doc]

```
# Genome denovo_assembling
mkdir 1.genome_denovo_assembling
cd 1.genome_denovo_assembling

mkdir  00.raw_data
cd 00.raw_data/
gzip -dc lib270/Reads.M4M24.wHADPI046654-23_1.fq.gz > A.1.fastq
gzip -dc lib270/Reads.M4M24.wHADPI046654-23_2.fq.gz > A.2.fastq
gzip -dc lib800/Reads.M4M24.wHAMPI046651-43_1.fq.gz >  B.1.fastq
gzip -dc lib800/Reads.M4M24.wHAMPI046651-43_2.fq.gz >  B.2.fastq
gzip -dc lib6k/Reads.M4M24.WHFUNuaiDCAADLAAPEI-32_1.fq.gz > C.1.fastq
gzip -dc lib6k/Reads.M4M24.WHFUNuaiDCAADLAAPEI-32_2 > C.2.fastq
gzip -dc lib6k/Reads.M4M24.WHFUNuaiDCAADLAAPEI-32_2.fq.gz > C.2.fastq
cd ..

mkdir 01.trimmomatic
cd 01.trimmomatic
/opt/sysoft/jre1.8.0_45/bin/java -jar /opt/biosoft/Trimmomatic-0.33/trimmomatic-0.33.jar PE -threads 24 ../00.raw_data/A.1.fastq ../00.raw_data/A.2.fastq A.1.fastq A.1.unpaired.fastq A.2.fastq A.2.unpaired.fastq ILLUMINACLIP:/opt/biosoft/Trimmomatic-0.33/adapters/TruSeq3-PE.fa:2:30:10 LEADING:3 TRAILING:3 SLIDINGWINDOW:4:15 MINLEN:50 TOPHRED33
/opt/sysoft/jre1.8.0_45/bin/java -jar /opt/biosoft/Trimmomatic-0.33/trimmomatic-0.33.jar PE -threads 24 ../00.raw_data/B.1.fastq ../00.raw_data/B.2.fastq B.1.fastq B.1.unpaired.fastq B.2.fastq B.2.unpaired.fastq ILLUMINACLIP:/opt/biosoft/Trimmomatic-0.33/adapters/TruSeq3-PE.fa:2:30:10 LEADING:3 TRAILING:3 SLIDINGWINDOW:4:15 MINLEN:50 TOPHRED33
/opt/sysoft/jre1.8.0_45/bin/java -jar /opt/biosoft/Trimmomatic-0.33/trimmomatic-0.33.jar PE -threads 24 ../00.raw_data/C.1.fastq ../00.raw_data/C.2.fastq C.1.fastq C.1.unpaired.fastq C.2.fastq C.2.unpaired.fastq ILLUMINACLIP:/opt/biosoft/Trimmomatic-0.33/adapters/TruSeq3-PE.fa:2:30:10 LEADING:3 TRAILING:3 SLIDINGWINDOW:4:15 MINLEN:36 TOPHRED33
cd ..

mkdir 02.ABYSS
cd 02.ABYSS
ln -s ../01.trimmomatic/?.?.fastq ./
source ~/.bashrc.mpich
source ~/.bashrc.gcc
/opt/biosoft/abyss-2.0.2/bin/abyss-pe np=24 k=81 name=mcra lib='pe1 pe2' mp=mp1 pe1='A.1.fastq A.2.fastq' pe2='B.1.fastq B.2.fastq' mp1='C.1.fastq C.2.fastq'
../scripts/genome_seq_clear.pl --seq_prefix abyss mcra-scaffolds.fa > abyss.fasta
cd ..

mkdir 03.insertSize
cd 03.insertSize
ln -s ../00.raw_data/?.?.fastq ./
../scripts/fasta2agp.pl ../02.ABYSS/abyss.fasta > contigs.fasta 2> /dev/null
../scripts/genome_seq_clear.pl --seq_prefix contig contigs.fasta > 11; mv 11 contigs.fasta
bowtie2-build contigs.fasta genome
bowtie2 -x genome -p 24 -1 A.1.fastq -2 A.2.fastq -S A.sam 2> A.bowtie2.log
bowtie2 -x genome -p 24 -1 B.1.fastq -2 B.2.fastq -S B.sam 2> B.bowtie2.log
bowtie2 -x genome -p 24 -1 C.1.fastq -2 C.2.fastq -S C.sam 2> C.bowtie2.log
../scripts/sam_getInsertSize.pl A.sam 150 1000
#227.06854184559	34.697914577772
../scripts/sam_getInsertSize.pl B.sam 300 2000
#745.562356920725	30.8249066356079
../scripts/sam_getInsertSize.pl C.sam 1000 20000
#6044.57434952794	571.534607803083
cd ..

mkdir 04.ALLPATHSLG
cd 04.ALLPATHSLG
ln -s ../01.trimmomatic/?.?.fastq ./
source ~/.bashrc.gcc
echo 'library_name, project_name, organism_name, type, paired, frag_size, frag_stddev, insert_size, insert_stddev, read_orientation, genomic_start, genomic_end
PE1_270bp, M04M24.genome, M.importuna, fragment, 1, 227, 35, , , inward, 0, 0
PE2_800bp, M04M24.genome, M.importuna, fragment, 1, 746, 31, , , inward, 0, 0
MP1_6kbp, M04M24.genome, M.importuna, jumping, 1, , , 6045, 572, outward, 0, 0' > in_libs.csv
echo 'group_name, library_name, file_name
PE1, PE1_270bp, A.?.fastq
PE2, PE2_800bp, B.?.fastq
MP1, MP1_6kbp, C.?.fastq' > in_groups.csv
ulimit -s 100000
mkdir -p M04M24.genome/data
PrepareAllPathsInputs.pl \
  DATA_DIR=$PWD/M04M24.genome/data \
  PLOIDY=1 \
  IN_GROUPS_CSV=in_groups.csv \
  IN_LIBS_CSV=in_libs.csv \
  OVERWRITE=True \
  | tee prepare.out
RunAllPathsLG \
  PRE=$PWD \
  REFERENCE_NAME=M04M24.genome \
  DATA_SUBDIR=data \
  RUN=run \
  SUBDIR=test \
  OVERWRITE=True \
  VAPI_WARN_ONLY=True \
  | tee -a assemble.out
EfastaToFasta HEAD=M04M24.genome/data/run/ASSEMBLIES/test/final.assembly SPLIT_DIR=scaffolds IUPAC=False
EfastaToFasta HEAD=M04M24.genome/data/run/ASSEMBLIES/test/final.contigs SPLIT_DIR=contigs IUPAC=False
cat contigs/*.fsa > allpathslg.contigs.fata
perl -p -i -e 's/n/N/g' scaffolds/*.fsa
cat scaffolds/*.fsa > allpathslg.scaffolds.fasta
cd ..

mkdir 05.GapCloser
cd 05.GapCloser
ln -s ../04.ALLPATHSLG/allpathslg.scaffolds.fasta genome.fa
ln -s ../01.trimmomatic/?.?.fastq ./
echo "max_rd_len=150
[LIB]
avg_ins=227
reverse_seq=0
asm_flags=1
rd_len_cutoff=150
rank=1
pair_num_cutoff=3
map_len=32
q1=$PWD/A.1.fastq
q2=$PWD/A.2.fastq
[LIB]
avg_ins=746
reverse_seq=0
asm_flags=3
rd_len_cutoff=150
rank=2
pair_num_cutoff=3
map_len=32
q1=$PWD/B.1.fastq
q2=$PWD/B.2.fastq
[LIB]
avg_ins=6045
reverse_seq=1
asm_flags=2
rd_len_cutoff=50
rank=3
pair_num_cutoff=5
map_len=35
q1=$PWD/C.1.fastq
q2=$PWD/C.2.fastq" > config.txt
GapCloser -a genome.fa -b config.txt -o gapcloser.fa -l 150 -t 40
cd ..

mkdir 06.SSPACE
cd 06.SSPACE
ln -s ../05.GapCloser/gapcloser.fa genome.fasta
bowtie2-build genome.fasta genome
bowtie2 -x genome -p 80 -U ../01.trimmomatic/B.1.fastq -S B1.sam --score-min L,-0.3,-0.3 2> bowtie2.B1.log
bowtie2 -x genome -p 80 -U ../01.trimmomatic/B.2.fastq -S B2.sam --score-min L,-0.3,-0.3 2> bowtie2.B2.log
bowtie2 -x genome -p 80 -U ../01.trimmomatic/C.1.fastq -S C1.sam --score-min L,-0.3,-0.3 2> bowtie2.C1.log
bowtie2 -x genome -p 80 -U ../01.trimmomatic/C.2.fastq -S C2.sam --score-min L,-0.3,-0.3 2> bowtie2.C2.log
../scripts/sspace_sam2tab.pl B1.sam B2.sam > sspace_in.B.tab 2> sspace_sam2tab.B.log
../scripts/sspace_sam2tab.pl C1.sam C2.sam > sspace_in.C.tab 2> sspace_sam2tab.C.log
echo -e "Lib2\tTAB\tsspace_in.B.tab\t746\t0.13\tFR
Lib2\tTAB\tsspace_in.C.tab\t6045\t0.29\tRF" > library.txt
SSPACE_Standard_v3.0.pl -l library.txt -s genome.fasta -x 0 -T 24 -b SSPACE
../scripts/genome_seq_clear.pl --seq_prefix sspace --min_length 500 SSPACE/SSPACE.final.scaffolds.fasta > sspace.fasta
cd ..

mkdir 07.GapCloser
cd 07.GapCloser
ln -s ../06.SSPACE/sspace.fasta genome.fa
ln -s ../01.trimmomatic/?.?.fastq ./
ln -s ../05.GapCloser/config.txt ./
GapCloser -a genome.fa -b config.txt -o gapcloser.fa -l 150 -t 40
../scripts/genome_seq_clear.pl --seq_prefix gapcloser --min_length 500 gapcloser.fa > gapcloser.fasta
cd ..

mkdir 08.pilon
cd 08.pilon
ln -s ../07.GapCloser/gapcloser.fasta genome.fasta
ln -s ../01.trimmomatic/?.?.fastq ./
bowtie2-build genome.fasta genome
bowtie2 -x genome -p 80 -1 A.1.fastq -2 A.2.fastq --score-min L,-0.3,-0.3 -I 150 -X 600 --fr --rg-id A --rg "PL:ILLUMINA" --rg "SM:A" -S A.sam 2> A.bowtie2.log
bowtie2 -x genome -p 80 -1 B.1.fastq -2 B.2.fastq --score-min L,-0.3,-0.3 -I 300 -X 1200 --fr --rg-id B --rg "PL:ILLUMINA" --rg "SM:B" -S B.sam 2> B.bowtie2.log
bowtie2 -x genome -p 80 -1 C.1.fastq -2 C.2.fastq --score-min L,-0.3,-0.3 -I 4000 -X 8000 --rf --rg-id C --rg "PL:ILLUMINA" --rg "SM:C" -S C.sam 2> C.bowtie2.log
samtools sort -@ 24 -o A.bam -O BAM A.sam
samtools sort -@ 24 -o B.bam -O BAM B.sam
samtools sort -@ 24 -o C.bam -O BAM C.sam
samtools index A.bam
samtools index B.bam
samtools index C.bam
samtools faidx genome.fasta
/opt/sysoft/jre1.8.0_45/bin/java -Xmx100G -jar /opt/biosoft/pilon/pilon-1.22.jar --genome genome.fasta --frags A.bam --frags B.bam --jumps C.bam --changes --vcf
../scripts/genome_seq_clear.pl --seq_prefix MI_M04M24_Scaffold --min_length 500 pilon.fasta > ../genome.fasta
cd ..


# Genome annotation
mkdir 2.genome_annotation
cd 2.genome_annotation
ln -s ../1.genome_denovo_assembling/genome.fasta ./


mkdir a.repeat_analysis
cd a.repeat_analysis
fasta_no_blank.pl ../genome.fasta > genome.fasta
mkdir repeatMasker/
/opt/biosoft/RepeatMasker/RepeatMasker -pa 80 -e ncbi -species fungi -dir repeatMasker/ -gff genome.fasta

mkdir repeatModeler
cd repeatModeler
/opt/biosoft/RepeatModeler-open-1.0.9/BuildDatabase -name species -engine ncbi ../genome.fasta
/opt/biosoft/RepeatModeler-open-1.0.9/RepeatModeler -engine ncbi -pa 80 -database species
/opt/biosoft/RepeatMasker/RepeatMasker -pa 80 -e ncbi -lib RM*/consensi.fa.classified -dir ./ -gff ../genome.fasta
cd ..

merge_repeatMasker_out.pl repeatMasker/genome.fasta.out repeatModeler/genome.fasta.out > genome.repeat.stats
maskedByGff.pl genome.repeat.gff3 genome.fasta > genome.hardmaskN.fasta
maskedByGff.pl --mask_type softmask genome.repeat.gff3 genome.fasta > genome.softmask.fasta
cd ..


mkdir b.non_coding_gene
cd b.non_coding_gene

cd ..


mkdir c.geta
cd c.geta
ln -s ../c.protein_coding_gene_prediction/reads.* ./
ln -s ../c.protein_coding_gene_prediction/homolog.fasta ./
ln -s ../genome.fasta ./
ln -s ../a.repeat_analysis/repeatModeler/RM_71362.TueMar201755182018/consensi.fa.classified ./
geta.pl --RM_species fungi --genome genome.fasta -1 reads.1.fastq -2 reads.2.fastq --protein homolog.fasta --augustus_species morchella_importuna_M04M24_geta --RM_lib consensi.fa.classified --cpu 160 --pfam_db /opt/biosoft/hmmer-3.1b2/Pfam-AB.hmm --gene_prefix MIM04M24Gene &> geta.log
cd ..


mkdir c.protein_coding_gene_prediction
cd c.protein_coding_gene_prediction
hisat2-build ../genome.fasta genome
# homolog protein sequences: homolog.fasta
# Tuber melanosporum (Perigord truffle) https://www.ncbi.nlm.nih.gov/genome/2215
# wget ftp://ftp.ncbi.nlm.nih.gov/genomes/all/GCF/000/151/645/GCF_000151645.1_ASM15164v1/GCF_000151645.1_ASM15164v1_protein.faa.gz
# gzip -dc GCF_000151645.1_ASM15164v1_protein.faa.gz > homolog.fasta
# perl -p -i -e 's/^(>\w+).*/$1/' homolog.fasta
# RNA-Seq Paired-end reads: reads.1.fastq reads.2.fastq
hisat2 -p 80 -x genome -1 reads.1.fastq -2 reads.2.fastq --dta --min-intronlen 20 --max-intronlen 20000 --score-min L,0.0,-0.4 -S hisat2.sam 2> hisat2.log
samtools sort -@ 40 -o hisat2.bam -O BAM hisat2.sam
ln -s ../a.repeat_analysis/genome.softmask.fasta genome.fasta
export AUGUSTUS_CONFIG_PATH=/opt/biosoft/augustus-3.2.3/config/
export AUGUSTUS_BIN_PATH=/opt/biosoft/augustus-3.2.3/bin/
export AUGUSTUS_SCRIPTS_PATH=/opt/biosoft/augustus-3.2.3/scripts/
export GENEMARK_PATH=/opt/biosoft/gm_et_linux_64_V4.33/gmes_petap/
export BAMTOOLS_PATH=/opt/biosoft/bamtools/bin/
export SAMTOOLS_PATH=/opt/biosoft/samtools-1.4/bin/
export ALIGNMENT_TOOL_PATH=/opt/biosoft/gth-1.7.0-Linux_x86_64-64bit/bin
#wget https://raw.githubusercontent.com/lfaino/LoReAn/master/third_party/conf_files/extrinsic.M.RM.E.W.P.cfg -O /opt/biosoft/augustus-3.2.3/config/extrinsic/extrinsic.M.RM.E.W.P.cfg
PATH=/opt/biosoft/BRAKER_v2.0.5_m/:$PATH
braker.pl --species=morchella_importuna_M04M24 --genome=genome.fasta --bam=hisat2.bam --prot_seq=homolog.fasta --softmasking --cores 80 --prg=gth --gth2traingenes --extrinsicCfgFile=/opt/biosoft/BRAKER_v2.0.5/extrinsic.M.RM.E.W.P.cfg
/opt/biosoft/augustus-3.2.3/scripts/gtf2gff.pl --gff3 < braker/morchella_importuna_M04M24/augustus.hints.gff --printExon --out=braker.gff3
gff3_clear.pl --prefix MIM04M24Gene braker.gff3 > genome.gff3
gff3ToGtf.pl ../genome.fasta genome.gff3 > genome.gtf
bestGeneModels.pl genome.gff3 > genome.bestGeneModels.gff3 2> genome.bestGeneModels.stats
gff3ToGtf.pl ../genome.fasta genome.bestGeneModels.gff3 > genome.bestGeneModels.gtf
eukaryotic_gene_model_statistics.pl genome.bestGeneModels.gtf ../genome.fasta out > eukaryotic_gene_model_statistics.txt
python /opt/biosoft/BUSCO_V3.0.2b/scripts/run_BUSCO.py -i out.pep.fasta -c 80 -o BUSCO_result -m proteins -l /opt/biosoft/BUSCO_V3.0.2b/fungi_odb9/

mkdir for_webApollo
cd for_webApollo
ln -s ../genome.fasta ./
ln -s ../genome.gff3 ./
bam2wig ../hisat2.bam > hisat2.wig
cal_seq_length.pl genome.fasta > chrom.sizes
wigToBigWig.pl hisat2.wig chrom.sizes hisat2.bw
# WebApollo²¿Êð: run.sh
cd ..


mkdir d.functional_annotation
cd d.functional_annotation
perl -pe 's/\*$//' ../c.protein_coding_gene_prediction/out.pep.fasta > proteins.fasta

mkdir nr
cd nr
perl -pe 's/-outfmt/-max_target_seqs 20 -outfmt/' ../../../scripts/blast.pl > blast.pl
perl blast.pl blastp nr ../proteins.fasta 1e-5 80 nr 5
../../../scripts/parsing_blast_result.pl nr.xml 20 1e-5 0.2 > nr.tab
../../../scripts/species_distribution_by_uniq_species_hits.pl nr.tab > species_distribution_by_uniq_species_hits.txt
cd ..

mkdir swissprot
cd swissprot
perl -pe 's/-outfmt/-max_target_seqs 20 -outfmt/' ../../../scripts/blast.pl > blast.pl
perl blast.pl blastp uniprot_sprot ../proteins.fasta 1e-5 80 uniprot_sprot 5
../../../scripts/parsing_blast_result.pl uniprot_sprot.xml 20 1e-5 0.2 > uniprot_sprot.tab
cd ..

mkdir COG
cd COG
../../../scripts/blast.pl blastp cog ../proteins.fasta 1e-5 80 cog 5
../../../scripts/cog_from_xml.pl cog.xml
../../../scripts/cog_R.pl cog_cog_class_annot.xls cog morchella_importuna_M04M24
../../../scripts/blast.pl blastp kog ../proteins.fasta 1e-5 80 kog 5
../../../scripts/kog_from_xml.pl kog.xml
../../../scripts/kog_R.pl kog_kog_class_annot.xls kog morchella_importuna_M04M24
cd ..

mkdir CDD
cd CDD
../../../scripts/split_fasta_file_averagely.pl ../proteins.fasta 3
# ½«ÐòÁÐÌá½»µ½https://www.ncbi.nlm.nih.gov/Structure/bwrpsb/bwrpsb.cgi
# QM3-qcdsearch-161483B58B5EEFA8
# QM3-qcdsearch-3BEC234477C47075
# QM3-qcdsearch-B82AFCF50DDE6B3
# mi0_hitdata.txt
cat *.txt | perl -e 'while (<>) { if (m/^#/ or m/^\s*$/) { next; } elsif (m/^Query/) { print}  else { s/.*?>//; $out{$_} = 1; } } foreach (sort keys %out) { print } ' | uniq > CDD.tab
rm *.fasta *.txt
cd ..

mkdir interpro
cd interpro
../../../scripts/interProScan5.pl ../proteins.fasta chenllianfu@gmail.com interpro5 30
cat interpro5/*.tsv.txt > interpro5.tsv
cd ..

mkdir go
cd go
# b2g4pipe
cd b2g4pipe
../../../../scripts/split_blast_xml.pl ../../nr/nr.xml 20
for i in `ls ??.xml`
do
    i=${i/.xml/}
    echo "java -cp *:ext/*: es.blast2go.prog.B2GAnnotPipe -in $i.xml -out go_$i -annot -dat -annex"
done > command.blast2go.list
ParaFly -c command.blast2go.list -CPU 20
cd ..
cat b2g4pipe/go_??.annot | grep GO | cut -f 1,2 > go.annot
../../../scripts/merge_interpro_to_go.pl go.annot ../interpro/interpro5.tsv > aa; mv aa go.annot
cd ..

mkdir kaas
cd kaas
# ÔÚ http://www.genome.jp/kaas-bin/kaas_main ÍøÒ³¹¤¾ßÖÐÌá½»ÐòÁÐ½øÐÐ×¢ÊÍ

cd ..

mkdir tf
cd tf
../../../scripts/interpro2tf.pl ../interpro/interpro5.tsv
cd ..
```
