# Supplementary material for: Opposite Polarity Monospore Genome De Novo Sequencing and Comparative Analysis Reveal the Possible Heterothallic Life Cycle of Morchella importuna
Source: Int J Mol Sci. 2018 Aug 25;19(9):2525. doi: 10.3390/ijms19092525 (PMC6164635; doi:10.3390/ijms19092525)
Supplement: Supplementary file 1 [file ijms-19-02525-s001.zip › Supplement/Supplementary Material 2.doc]

\# Genome de novo assembly

mkdir 1.genome_denovo_assembling

cd 1.genome_denovo_assembling

mkdir 00.raw_data

cd 00.raw_data

gzip -dc ../../0.initial_data/FCHCF3FBBXX_L6_wHADPI033900-102_1.fq.gz > A.1.fastq

gzip -dc ../../0.initial_data/FCHCF3FBBXX_L6_wHADPI033900-102_2.fq.gz > A.2.fastq

gzip -dc ../../0.initial_data/FCHCF3FBBXX_L8_wHADPI033901-102_1.fq.gz > B.1.fastq

gzip -dc ../../0.initial_data/FCHCF3FBBXX_L8_wHADPI033901-102_2.fq.gz > B.2.fastq

for i in `ls ../../0.initial_data/FC*AAPEI*_1.fq.gz`

do

gzip -dc $i >> C.1.fastq

done

for i in `ls ../../0.initial_data/FC*AAPEI*_2.fq.gz`

do

gzip -dc $i >> C.2.fastq

done

cd ..

mkdir 01.FASTQC

cd 01.FASTQC

mkdir raw_data

/opt/biosoft/FastQC/fastqc -t 10 -o ./raw_data ../00.raw_data/*

cd ..

mkdir 02.NGSQCToolkit

cd 02.NGSQCToolkit

perl -pe 's/\n*$/\n/; s/^>.*\n//;' /opt/biosoft/Trimmomatic-0.33/adapters/TruSeq3-PE.fa > adaptor.txt

/opt/biosoft/NGSQCToolkit_v2.3.3/QC/IlluQC_PRLL.pl -l 60 -s 13 -c 20 -o A -pe ../00.raw_data/A.1.fastq ../00.raw_data/A.2.fastq adaptor.txt 5

/opt/biosoft/NGSQCToolkit_v2.3.3/QC/IlluQC_PRLL.pl -l 60 -s 13 -c 20 -o B -pe ../00.raw_data/B.1.fastq ../00.raw_data/B.2.fastq adaptor.txt 5

/opt/biosoft/NGSQCToolkit_v2.3.3/QC/IlluQC_PRLL.pl -l 60 -s 13 -c 20 -o C -pe ../00.raw_data/C.1.fastq ../00.raw_data/C.2.fastq adaptor.txt 5

ln -s A/A.1.fastq_filtered A.1.fastq

ln -s A/A.2.fastq_filtered A.2.fastq

ln -s B/B.1.fastq_filtered B.1.fastq

ln -s B/B.2.fastq_filtered B.2.fastq

ln -s C/C.1.fastq_filtered C.1.fastq

ln -s C/C.2.fastq_filtered C.2.fastq

cd ..

mkdir 03.BLESS

cd 03.BLESS

cat ../02.NGSQCToolkit/A.1.fastq ../02.NGSQCToolkit/B.1.fastq > AB.1.fastq

cat ../02.NGSQCToolkit/A.2.fastq ../02.NGSQCToolkit/B.2.fastq > AB.2.fastq

bless -read1 AB.1.fastq -read2 AB.2.fastq -kmerlength 21 -prefix AB -max_mem 64 -smpthread 40

bless -read1 ../02.NGSQCToolkit/A.1.fastq -read2 ../02.NGSQCToolkit/A.2.fastq -kmerlength 21 -prefix A_notrim -load AB -max_mem 40 -notrim -smpthread 20

bless -read1 ../02.NGSQCToolkit/B.1.fastq -read2 ../02.NGSQCToolkit/B.2.fastq -kmerlength 21 -prefix B_notrim -load AB -max_mem 40 -notrim -smpthread 20

bless -read1 ../02.NGSQCToolkit/C.1.fastq -read2 ../02.NGSQCToolkit/C.2.fastq -kmerlength 21 -prefix C_notrim -load AB -max_mem 40 -notrim -smpthread 20

bless -read1 ../02.NGSQCToolkit/A.1.fastq -read2 ../02.NGSQCToolkit/A.2.fastq -kmerlength 21 -prefix A -load AB -max_mem 40 -smpthread 20

bless -read1 ../02.NGSQCToolkit/B.1.fastq -read2 ../02.NGSQCToolkit/B.2.fastq -kmerlength 21 -prefix B -load AB -max_mem 40 -smpthread 20

bless -read1 ../02.NGSQCToolkit/C.1.fastq -read2 ../02.NGSQCToolkit/C.2.fastq -kmerlength 21 -prefix C -load AB -max_mem 40 -smpthread 20

cd ..

mkdir 04.FindErrors

cd 04.FindErrors

ErrorCorrectReads.pl PHRED_ENCODING=33 READS_OUT=fragment FILL_FRAGMENTS=1 KEEP_KMER_SPECTRA=1 PAIRED_READS_A_IN=../03.BLESS/AB.1.fastq PAIRED_READS_B_IN=../03.BLESS/AB.2.fastq PLOIDY=1 PAIRED_SEP=-30 PAIRED_STDEV=30 &> ErrorCorrectReads.log

cd fragment.fastq.kspec

perl -p -i -e 's/(my \@fns.*)/$1\n\@fns = \(\"frag_reads_filt.25mer.kspec\", \"frag_reads_edit.24mer.kspec\", \"frag_reads_corr.25mer.kspec\"\);/' /opt/biosoft/ALLPATHS-LG/bin/KmerSpectrumPlot.pl

KmerSpectrumPlot.pl SPECTRA=1

perl -p -i -e 's/\@fns = \(\"frag_reads_filt.25mer.kspec\", \"frag_reads_edit.24mer.kspec\", \"frag_reads_corr.25mer.kspec\"\);\n//' /opt/biosoft/ALLPATHS-LG/bin/KmerSpectrumPlot.pl

convert kmer_spectrum.cumulative_frac.log.lin.eps kmer_spectrum.cumulative_frac.log.lin.png

convert kmer_spectrum.distinct.lin.lin.eps kmer_spectrum.distinct.lin.lin.png

convert kmer_spectrum.distinct.log.log.eps kmer_spectrum.distinct.log.log.png

cd ..

mkdir 05.GCE

cd 05.GCE

ls $PWD/../02.NGSQCToolkit/A.?.fastq > reads.list

ls $PWD/../02.NGSQCToolkit/B.?.fastq >> reads.list

/opt/biosoft/gce-1.0.0/kmerfreq/kmer_freq_hash/kmer_freq_hash -k 21 -l reads.list -a 10 -d 10 -t 80 -i 50000000 -o 0 -p M04M26 &> kmer_freq.log

/opt/biosoft/gce-1.0.0/gce -f M04M26.freq.stat -c -g -m 1 -D 8 -b 1 > M04M26.table 2> M04M26.log

cd ..

mkdir 06.Edena

cd 06.Edena

ln -s ../03.BLESS/A_notrim.1.corrected.fastq A.1.fastq

ln -s ../03.BLESS/A_notrim.2.corrected.fastq A.2.fastq

ln -s ../03.BLESS/B_notrim.1.corrected.fastq B.1.fastq

ln -s ../03.BLESS/B_notrim.2.corrected.fastq B.2.fastq

edena -nThreads 80 -DRpairs A.1.fastq A.2.fastq B.1.fastq B.2.fastq -minOverlap 50

for ((i=50; i<=140; i=i+10))

do

echo "edena -e out.ovl -p out_OC$i -overlapCutoff $i"

done > command.edena.list

ParaFly -c command.edena.list -CPU 10

\# 当-overlapCutoff参数选择80的时候，contig N50值最大，为15649

ln -s out_OC80_contigs.fasta edena.fasta

cd ..

mkdir 07.InsertSize

cd 07.InsertSize

bowtie2-build ../06.Edena/edena.fasta edena

bowtie2 -p 80 -x edena -u 1000000 -1 ../02.NGSQCToolkit/A.1.fastq -2 ../02.NGSQCToolkit/A.2.fastq -S A.sam 2> A.bowtie2.log

bowtie2 -p 80 -x edena -u 1000000 -1 ../02.NGSQCToolkit/B.1.fastq -2 ../02.NGSQCToolkit/B.2.fastq -S B.sam 2> B.bowtie2.log

bowtie2 -p 80 -x edena -u 1000000 --rf -I 2000 -X 20000 -1 ../02.NGSQCToolkit/C.1.fastq -2 ../02.NGSQCToolkit/C.2.fastq -S C.sam 2> C.bowtie2.log

samtools sort -@ 20 -m 4G A.sam A

samtools sort -@ 20 -m 4G B.sam B

samtools sort -@ 20 -m 4G C.sam C

/opt/sysoft/jre1.8.0_45/bin/java -jar /opt/biosoft/picard-tools-2.5.0/picard.jar CollectInsertSizeMetrics I=A.bam O=A.insertSize.txt H=A.insertSize.pdf W=600

/opt/sysoft/jre1.8.0_45/bin/java -jar /opt/biosoft/picard-tools-2.5.0/picard.jar CollectInsertSizeMetrics I=B.bam O=B.insertSize.txt H=B.insertSize.pdf W=600

/opt/sysoft/jre1.8.0_45/bin/java -jar /opt/biosoft/picard-tools-2.5.0/picard.jar CollectInsertSizeMetrics I=C.bam O=C.insertSize.txt H=C.insertSize.pdf W=15000

cd ..

mkdir 08.AllPathsLG

cd 08.AllPathsLG

ln -s ../02.NGSQCToolkit/*.fastq .

ln -s ../../0.initial_data/PacBio.subreads.fastq ./

echo 'library_name, project_name, organism_name, type, paired, frag_size, frag_stddev, insert_size, insert_stddev, read_orientation, genomic_start, genomic_end

PE1_270bp, M04M26.genome, M.crasspies, fragment, 1, 247, 33, , , inward, 0, 0

PE2_270bp, M04M26.genome, M.crasspies, fragment, 1, 230, 32, , , inward, 0, 0

MP1_10kbp, M04M26.genome, M.crasspies, jumping, 1, , , 11002, 1956, outward, 0, 0

Pacbio_20kbp, M04M26.genome, M.crasspies, long, 0, , , , , , 0, 0' > in_libs.csv

echo 'group_name, library_name, file_name

PE1, PE1_270bp, A.?.fastq

PE2, PE2_270bp, B.?.fastq

MP1, MP1_10kbp, C.?.fastq

Pacbio, Pacbio_20kbp, PacBio.subreads.fastq' > in_groups.csv

source ~/.bashrc.gcc

ulimit -s 100000

mkdir -p M04M26.genome/data

PrepareAllPathsInputs.pl \

DATA_DIR=$PWD/M04M26.genome/data \

PLOIDY=1 \

IN_GROUPS_CSV=in_groups.csv \

IN_LIBS_CSV=in_libs.csv \

OVERWRITE=True \

| tee prepare.out

RunAllPathsLG \

PRE=$PWD \

REFERENCE_NAME=M04M26.genome \

DATA_SUBDIR=data \

RUN=run \

SUBDIR=test \

OVERWRITE=True \

VAPI_WARN_ONLY=True \

| tee -a assemble.out

EfastaToFasta HEAD=M04M26.genome/data/run/ASSEMBLIES/test/final.assembly SPLIT_DIR=scaffolds IUPAC=False

EfastaToFasta HEAD=M04M26.genome/data/run/ASSEMBLIES/test/final.contigs SPLIT_DIR=contigs IUPAC=False

cat contigs/*.fsa > allpathslg.contigs.fata

perl -p -i -e 's/n/N/g' scaffolds/*.fsa

cat scaffolds/*.fsa > allpathslg.scaffolds.fasta

../../scripts/genome_seq_clear.pl --seq_prefix ALLPATHSLG --min_length 500 allpathslg.scaffolds.fasta > 11; mv 11 allpathslg.scaffolds.fasta

cd ..

mkdir 09.LoRDEC

cd 09.LoRDEC

pls2fasta ../../0.initial_data/Pacbio_M04M26_Cell01/Analysis_Results/m161008_091827_42208_c101104282550000001823260104261775_s1_p0.bas.h5 Pacbio_M04M26_Cell01.fastq -trimByRegion -fastq

pls2fasta ../../0.initial_data/Pacbio_M04M26_Cell02/Analysis_Results/m161031_174628_42208_c101103212550000001823259404261707_s1_p0.bas.h5 Pacbio_M04M26_Cell02.fastq -trimByRegion -fastq

lordec-build-SR-graph -T 24 -2 ../04.FindErrors/fragment.fastq -k 21 -s 4 -g illumina_21_4.h5

lordec-correct -2 illumina_21_4 -i Pacbio_M04M26_Cell01.fastq -k 21 -s 4 -T 32 -o Pacbio_M04M26_Cell01.fasta &> Pacbio_M04M26_Cell01.log

lordec-correct -2 illumina_21_4 -i Pacbio_M04M26_Cell02.fastq -k 21 -s 4 -T 32 -o Pacbio_M04M26_Cell02.fasta &> Pacbio_M04M26_Cell02.log

lordec-trim -i Pacbio_M04M26_Cell01.fasta -o Pacbio_M04M26_Cell01.fasta.trim

lordec-trim-split -i Pacbio_M04M26_Cell01.fasta -o Pacbio_M04M26_Cell01.fasta.trim-split

lordec-trim -i Pacbio_M04M26_Cell02.fasta -o Pacbio_M04M26_Cell02.fasta.trim

lordec-trim-split -i Pacbio_M04M26_Cell02.fasta -o Pacbio_M04M26_Cell02.fasta.trim-split

cd ..

mkdir 10.DBG2OLC

cd 10.DBG2OLC

mkdir SparseAssembler

cd SparseAssembler

SparseAssembler LD 0 k 95 g 15 NodeCovTh 1 EdgeCovTh 0 GS 60000000 f ../../04.FindErrors/fragment.paired.A.fastq f ../../04.FindErrors/fragment.paired.B.fastq

cp Contigs.txt Contigs.txt.00

SparseAssembler LD 1 k 95 g 15 NodeCovTh 2 EdgeCovTh 1 GS 60000000 f ../../04.FindErrors/fragment.paired.A.fastq f ../../04.FindErrors/fragment.paired.B.fastq

cp Contigs.txt Contigs.txt.01

SparseAssembler LD 1 k 95 g 15 NodeCovTh 3 EdgeCovTh 2 GS 60000000 f ../../04.FindErrors/fragment.paired.A.fastq f ../../04.FindErrors/fragment.paired.B.fastq

cp Contigs.txt Contigs.txt.02

SparseAssembler LD 1 k 95 g 15 NodeCovTh 4 EdgeCovTh 3 GS 60000000 f ../../04.FindErrors/fragment.paired.A.fastq f ../../04.FindErrors/fragment.paired.B.fastq

cp Contigs.txt Contigs.txt.03

cp Contigs.txt.02 Contigs.txt

cd ..

mkdir DBG2OLC

cd DBG2OLC

DBG2OLC LD 0 k 17 AdaptiveTh 0.0001 KmerCovTh 2 MinOverlap 20 RemoveChimera 1 Contigs ../SparseAssembler/Contigs.txt f ../../09.LoRDEC/Pacbio_M04M26_Cell01.fastq f ../../09.LoRDEC/Pacbio_M04M26_Cell02.fastq

cp backbone_raw.fasta backbone_raw.fasta.00

DBG2OLC LD 1 k 17 AdaptiveTh 0.0075 KmerCovTh 2 MinOverlap 30 RemoveChimera 1 Contigs ../SparseAssembler/Contigs.txt f ../../09.LoRDEC/Pacbio_M04M26_Cell01.fastq f ../../09.LoRDEC/Pacbio_M04M26_Cell02.fastq

cp backbone_raw.fasta backbone_raw.fasta.01

DBG2OLC LD 1 k 17 AdaptiveTh 0.01 KmerCovTh 2 MinOverlap 30 RemoveChimera 1 Contigs ../SparseAssembler/Contigs.txt f ../../09.LoRDEC/Pacbio_M04M26_Cell01.fastq f ../../09.LoRDEC/Pacbio_M04M26_Cell02.fastq

cp backbone_raw.fasta backbone_raw.fasta.02

DBG2OLC LD 1 k 17 AdaptiveTh 0.012 KmerCovTh 2 MinOverlap 30 RemoveChimera 1 Contigs ../SparseAssembler/Contigs.txt f ../../09.LoRDEC/Pacbio_M04M26_Cell01.fastq f ../../09.LoRDEC/Pacbio_M04M26_Cell02.fastq

cp backbone_raw.fasta backbone_raw.fasta.03

DBG2OLC LD 1 k 17 AdaptiveTh 0.012 KmerCovTh 2 MinOverlap 50 RemoveChimera 1 Contigs ../SparseAssembler/Contigs.txt f ../../09.LoRDEC/Pacbio_M04M26_Cell01.fastq f ../../09.LoRDEC/Pacbio_M04M26_Cell02.fastq

cp backbone_raw.fasta backbone_raw.fasta.04

DBG2OLC LD 1 k 17 AdaptiveTh 0.011 KmerCovTh 2 MinOverlap 50 RemoveChimera 1 Contigs ../SparseAssembler/Contigs.txt f ../../09.LoRDEC/Pacbio_M04M26_Cell01.fastq f ../../09.LoRDEC/Pacbio_M04M26_Cell02.fastq

cd ..

mkdir Call_consensus

cd Call_consensus

source ~/.bashrc.pacbio

cp /opt/biosoft/DBG2OLC/utility/*.sh /opt/biosoft/DBG2OLC/utility/*.py ./

cp ../SparseAssembler/Contigs.txt ctg_pb.fasta

perl -e 'while (<>) {s/^\@/>/; print; $_ = <>; print; <>; <>;}' ../../09.LoRDEC/Pacbio_M04M26_Cell01.fastq >> ctg_pb.fasta

perl -e 'while (<>) {s/^\@/>/; print; $_ = <>; print; <>; <>;}' ../../09.LoRDEC/Pacbio_M04M26_Cell02.fastq >> ctg_pb.fasta

./split_and_run_sparc.sh ../DBG2OLC/backbone_raw.fasta ../DBG2OLC/DBG2OLC_Consensus_info.txt ctg_pb.fasta ./ 2 > cns_log.txt

cd ..

genome_seq_clear.pl --seq_prefix DBG2OLC Call_consensus/final_assembly.fasta > DBG2OLC.fasta

cd ..

mkdir 11.FALCON

cd 11.FALCON

perl -e 'while (<>) { if (/^>/) { print } else { tr/atcgn/ATCGN/; print } }' ../09.LoRDEC/Pacbio_M10M26_Cell01.fasta > raw_reads.fasta

perl -e 'while (<>) { if (/^>/) { print } else { tr/atcgn/ATCGN/; print } }' ../09.LoRDEC/Pacbio_M10M26_Cell02.fasta >> raw_reads.fasta

../../scripts/genome_seq_clear.pl --no_sort --no_rename --min_length 500 --line_length -1 raw_reads.fasta > 11; mv 11 raw_reads.fasta

echo "raw_reads.fasta" > input.fofn

echo '[General]

job_type = local

input_fofn = input.fofn

input_type = raw

length_cutoff = 5000

length_cutoff_pr = 5000

pa_DBsplit_option = -x500 -s50

pa_HPCdaligner_option = -v -B4 -k14 -T8 -t16 -e.70 -l500 -s500

falcon_sense_option = --output_multi --min_idt 0.70 --min_cov 2 --min_cov_aln 4 --min_n_read 4 --max_n_read 200 --n_core 2

pa_concurrent_jobs = 15

la_concurrent_jobs = 2

cns_concurrent_jobs = 40

sge_option_da = -pe smp 16 -q jobqueue

sge_option_la = -pe smp 8 -q jobqueue

sge_option_fc = -pe smp 80 -q jobqueue

sge_option_cns = -pe smp 16 -q jobqueue

ovlp_DBsplit_option = -x500 -s50

ovlp_HPCdaligner_option = -v -B4 -k20 -h60 -T8 -t32 -e.96 -l500 -s500

pda_concurrent_jobs = 15

pla_concurrent_jobs = 2

sge_option_pda = -pe smp 16 -q jobqueue

sge_option_pla = -pe smp 8 -q jobqueue

overlap_filtering_setting = --max_diff 100 --max_cov 200 --min_cov 2 --bestn 30 --n_core 40' > fc_run.cfg

cd /opt/biosoft/FALCON-integrate/

source env.sh

cd -

fc_run.py fc_run.cfg

../../scripts/genome_seq_clear.pl --seq_prefix FALCON 2-asm-falcon/p_ctg.fa > FALCON.fasta

cd ..

mkdir 12.Metassembler

cd 12.Metassembler

head -n 4000000 ../03.BLESS/C.1.corrected.fastq > C.1.fastq

head -n 4000000 ../03.BLESS/C.2.corrected.fastq > C.2.fastq

ln -s ../08.AllPathsLG/allpathslg.scaffolds.fasta ALLPATHSLG.fasta

ln -s ../10.DBG2OLC/DBG2OLC.fasta ./

ln -s ../11.FALCON/FALCON.fasta ./

metassemble2genomes.pl --genome1 $PWD/DBG2OLC.fasta --genome2 $PWD/FALCON.fasta --fastq1 C.1.fastq --fastq2 C.2.fastq --bowtie2_cpu 80 --mateAn_A 5000 --mateAn_B 17000 --nucmer_l 50 --nucmer_c 300 --out $PWD/first

../../scripts/genome_seq_clear.pl --seq_prefix first --min_length 500 first/Metassembly/QB.A/M1/QB.A.fasta > first.fasta

metassemble2genomes.pl --genome1 $PWD/first.fasta --genome2 $PWD/ALLPATHSLG.fasta --fastq1 C.1.fastq --fastq2 C.2.fastq --bowtie2_cpu 80 --mateAn_A 5000 --mateAn_B 17000 --nucmer_l 50 --nucmer_c 300 --out $PWD/second

../../scripts/genome_seq_clear.pl --seq_prefix metassembler --min_length 500 second/Metassembly/QB.A/M1/QB.A.fasta > metassembler.fasta

cd ..

mkdir 13.SSPACE

cd 13.SSPACE

head -n 8000000 ../03.BLESS/C.1.corrected.fastq > reads.1.fastq

head -n 8000000 ../03.BLESS/C.2.corrected.fastq > reads.2.fastq

ln -s ../12.Metassembler/metassembler.fasta genome.fasta

bowtie2-build genome.fasta genome

bowtie2 -x genome -1 reads.1.fastq -2 reads.2.fastq --score-min L,-0.3,-0.3 -S bowtie2.sam 2> bowtie2.log

../../scripts/sspace_sam2tab.pl bowtie2.sam bowtie2.tab &> /dev/null

perl -e 'while (<>) { chomp; @_ = split /\t/; @aa = ("$_[0]\t$_[1]\t$_[2]", $two = "$_[3]\t$_[4]\t$_[5]"); @aa = sort {$a cmp $b} @aa; $aa = join "\t", @aa; $hash{$aa} = 1; } foreach (keys %hash) { print "$_\n"; }' bowtie2.tab > bowtie2.rmDup.tab

../../scripts/sam_getInsertSize.pl bowtie2.sam 1000 20000

#10944.2431067225	1225.76738659717

echo -e "Lib1\tTAB\tbowtie2.rmDup.tab\t10944\t0.34\tRF" > library.txt

wc -l bowtie2.rmDup.tab

\# 980678

\# (980678*100/50922560)*(10944/100)=210.76	210.76/20=10.54

SSPACE_Standard_v3.0.pl -l library.txt -s genome.fasta -x 0 -k 11

../../scripts/genome_seq_clear.pl --seq_prefix SSPACE --min_length 500 standard_output/standard_output.final.scaffolds.fasta > SSPACE.fasta

cd ..

mkdir 14.finisherSC

cd 14.finisherSC

ln -s ../13.SSPACE/SSPACE.fasta contigs.fasta

ln -s ../11.FALCON/raw_reads.fasta ./

python /opt/biosoft/finishingTool/finisherSC.py -par 80 ./ /opt/biosoft/finishingTool/MUMmer3.23/

../../scripts/genome_seq_clear.pl --seq_prefix finisherSC --min_length 500 improved3.fasta > finisherSC.fasta

cd ..

mkdir 15.pilon

cd 15.pilon

ln -s ../03.BLESS/AB.1.fastq reads.1.fastq

ln -s ../03.BLESS/AB.2.fastq reads.2.fastq

ln -s ../14.finisherSC/finisherSC.fasta genome.fasta

bowtie2-build genome.fasta genome

bowtie2 -x genome -p 80 -1 reads.1.fastq -2 reads.2.fastq --score-min L,-0.3,-0.3 --rdg 10,1 --rfg 10,1 --rg-id sample --rg "PL:ILLUMINA" --rg "SM:sample" -S bowtie2.sam 2> bowtie2.log

samtools sort -@ 24 -o bowtie2.bam -O BAM bowtie2.sam

samtools index bowtie2.bam

samtools faidx genome.fasta

/opt/sysoft/jre1.8.0_45/bin/java -Xmx100G -jar /opt/biosoft/pilon/pilon-1.22.jar --genome genome.fasta --bam bowtie2.bam --changes --vcf --threads 80

../../scripts/genome_seq_clear.pl --seq_prefix pilon --min_length 500 pilon.fasta > pilon.01.fasta

bowtie2-build pilon.01.fasta genome

bowtie2 -x genome -p 80 -1 reads.1.fastq -2 reads.2.fastq --score-min L,-0.3,-0.3 --rdg 10,1 --rfg 10,1 --rg-id sample --rg "PL:ILLUMINA" --rg "SM:sample" -S bowtie2.sam 2> bowtie2.log

samtools sort -@ 24 -o bowtie2.bam -O BAM bowtie2.sam

samtools index bowtie2.bam

samtools faidx genome.fasta

/opt/sysoft/jre1.8.0_45/bin/java -Xmx100G -jar /opt/biosoft/pilon/pilon-1.22.jar --genome pilon.01.fasta --bam bowtie2.bam --changes --vcf --threads 80

../../scripts/genome_seq_clear.pl --seq_prefix pilon --min_length 500 pilon.fasta > pilon.02.fasta

cd ..

mkdir 16.quiver

cd 16.quiver

source /home/smrtanalysis/install/smrtanalysis_2.3.0.140936/etc/setup.sh

ln -s ../15.pilon/pilon.fasta genome.fasta

ls $PWD/../../0.initial_data/Pacbio_M04M26_Cell0*/*/*.bax.h5 > input.fofn

fofnToSmrtpipeInput.py input.fofn > input.xml

referenceUploader -c -f $PWD/genome.fasta -n genome -o morimp -p ./ --ploidy haploid

samtools faidx genome/sequence/genome.fasta

\# https://github.com/PacificBiosciences/GenomicConsensus/blob/master/doc/params-template.xml 中有个配置文件模板，设置其基因组数据库路径（$PWD/genome）即可。

\# params.xml

smrtpipe.py --params=params.xml xml:input.xml

gzip -dc data/consensus.fasta.gz > 11

../../scripts/genome_seq_clear.pl --seq_prefix quiver --min_length 500 11 > quiver.fasta; rm 11

cd ..

../scripts/genome_seq_clear.pl --seq_prefix Morimp01GS 16.quiver/quiver.fasta > Morchella_importuna_v1.0_GS.fasta

perl -p -i -e 'tr/atcgn/ATCGN/ unless m/^>/' Morchella_importuna_v1.0_GS.fasta

\# Genome annotation

mkdir 2.genome_annotation

cd 2.genome_annotation

ln -s ../1.genome_denovo_assembling/genome.fasta ./

mkdir a.repeat_analysis

cd a.repeat_analysis

fasta_no_blank.pl ../genome.fasta > genome.fasta

mkdir repeatMasker/

/opt/biosoft/RepeatMasker/RepeatMasker -pa 80 -e ncbi -species fungi -dir repeatMasker/ -gff genome.fasta

mkdir repeatModeler

cd repeatModeler

/opt/biosoft/RepeatModeler-open-1.0.9/BuildDatabase -name species -engine ncbi ../genome.fasta

/opt/biosoft/RepeatModeler-open-1.0.9/RepeatModeler -engine ncbi -pa 80 -database species

/opt/biosoft/RepeatMasker/RepeatMasker -pa 80 -e ncbi -lib RM*/consensi.fa.classified -dir ./ -gff ../genome.fasta

cd ..

merge_repeatMasker_out.pl repeatMasker/genome.fasta.out repeatModeler/genome.fasta.out > genome.repeat.stats

maskedByGff.pl genome.repeat.gff3 genome.fasta > genome.hardmaskN.fasta

maskedByGff.pl --mask_type softmask genome.repeat.gff3 genome.fasta > genome.softmask.fasta

cd ..

mkdir b.non_coding_gene

cd b.non_coding_gene

cd ..

mkdir c.geta

cd c.geta

ln -s ../c.protein_coding_gene_prediction/reads.* ./

ln -s ../c.protein_coding_gene_prediction/homolog.fasta ./

ln -s ../genome.fasta ./

ln -s ../a.repeat_analysis/repeatModeler/RM_43119.ThuMar221820432018/consensi.fa.classified ./

geta.pl --RM_species fungi --genome genome.fasta -1 reads.1.fastq -2 reads.2.fastq --protein homolog.fasta --augustus_species morchella_importuna_M04M26_geta --RM_lib consensi.fa.classified --cpu 160 --pfam_db /opt/biosoft/hmmer-3.1b2/Pfam-AB.hmm --gene_prefix MIM04M26Gene &> geta.log

cd ..

mkdir c.protein_coding_gene_prediction

cd c.protein_coding_gene_prediction

hisat2-build ../genome.fasta genome

\# homolog protein sequences: homolog.fasta

\# Tuber melanosporum (Perigord truffle) https://www.ncbi.nlm.nih.gov/genome/2215

\# wget ftp://ftp.ncbi.nlm.nih.gov/genomes/all/GCF/000/151/645/GCF_000151645.1_ASM15164v1/GCF_000151645.1_ASM15164v1_protein.faa.gz

\# gzip -dc GCF_000151645.1_ASM15164v1_protein.faa.gz > homolog.fasta

\# perl -p -i -e 's/^(>\w+).*/$1/' homolog.fasta

\# RNA-Seq Paired-end reads: reads.1.fastq reads.2.fastq

hisat2 -p 80 -x genome -1 reads.1.fastq -2 reads.2.fastq --dta --min-intronlen 20 --max-intronlen 20000 --score-min L,0.0,-0.4 -S hisat2.sam 2> hisat2.log

samtools sort -@ 40 -o hisat2.bam -O BAM hisat2.sam

ln -s ../a.repeat_analysis/genome.softmask.fasta genome.fasta

export AUGUSTUS_CONFIG_PATH=/opt/biosoft/augustus-3.2.3/config/

export AUGUSTUS_BIN_PATH=/opt/biosoft/augustus-3.2.3/bin/

export AUGUSTUS_SCRIPTS_PATH=/opt/biosoft/augustus-3.2.3/scripts/

export GENEMARK_PATH=/opt/biosoft/gm_et_linux_64_V4.33/gmes_petap/

export BAMTOOLS_PATH=/opt/biosoft/bamtools/bin/

export SAMTOOLS_PATH=/opt/biosoft/samtools-1.4/bin/

export ALIGNMENT_TOOL_PATH=/opt/biosoft/gth-1.7.0-Linux_x86_64-64bit/bin

#wget https://raw.githubusercontent.com/lfaino/LoReAn/master/third_party/conf_files/extrinsic.M.RM.E.W.P.cfg -O /opt/biosoft/augustus-3.2.3/config/extrinsic/extrinsic.M.RM.E.W.P.cfg

PATH=/opt/biosoft/BRAKER_v2.0.5_m/:$PATH

braker.pl --species=morchella_importuna_M04M26 --genome=genome.fasta --bam=hisat2.bam --prot_seq=homolog.fasta --softmasking --cores 80 --prg=gth --gth2traingenes --extrinsicCfgFile=/opt/biosoft/BRAKER_v2.0.5/extrinsic.M.RM.E.W.P.cfg

/opt/biosoft/augustus-3.2.3/scripts/gtf2gff.pl --gff3 < braker/morchella_importuna_M04M26/augustus.hints.gff --printExon --out=braker.gff3

gff3_clear.pl --prefix MIM04M26Gene braker.gff3 > genome.gff3

gff3ToGtf.pl ../genome.fasta genome.gff3 > genome.gtf

bestGeneModels.pl genome.gff3 > genome.bestGeneModels.gff3 2> genome.bestGeneModels.stats

gff3ToGtf.pl ../genome.fasta genome.bestGeneModels.gff3 > genome.bestGeneModels.gtf

eukaryotic_gene_model_statistics.pl genome.bestGeneModels.gtf ../genome.fasta out > eukaryotic_gene_model_statistics.txt

python /opt/biosoft/BUSCO_V3.0.2b/scripts/run_BUSCO.py -i out.pep.fasta -c 80 -o BUSCO_result -m proteins -l /opt/biosoft/BUSCO_V3.0.2b/fungi_odb9/

mkdir for_webApollo

cd for_webApollo

ln -s ../genome.fasta ./

ln -s ../genome.gff3 ./

bam2wig ../hisat2.bam > hisat2.wig

cal_seq_length.pl genome.fasta > chrom.sizes

wigToBigWig.pl hisat2.wig chrom.sizes hisat2.bw

\# WebApollo部署: run.sh

cd ..

mkdir d.functional_annotation

cd d.functional_annotation

perl -pe 's/\*$//' ../c.protein_coding_gene_prediction/out.pep.fasta > proteins.fasta

mkdir nr

cd nr

perl -pe 's/-outfmt/-max_target_seqs 20 -outfmt/' ../../../scripts/blast.pl > blast.pl

perl blast.pl blastp nr ../proteins.fasta 1e-5 80 nr 5

../../../scripts/parsing_blast_result.pl nr.xml 20 1e-5 0.2 > nr.tab

../../../scripts/species_distribution_by_uniq_species_hits.pl nr.tab > species_distribution_by_uniq_species_hits.txt

cd ..

mkdir swissprot

cd swissprot

perl -pe 's/-outfmt/-max_target_seqs 20 -outfmt/' ../../../scripts/blast.pl > blast.pl

perl blast.pl blastp uniprot_sprot ../proteins.fasta 1e-5 80 uniprot_sprot 5

../../../scripts/parsing_blast_result.pl uniprot_sprot.xml 20 1e-5 0.2 > uniprot_sprot.tab

cd ..

mkdir COG

cd COG

../../../scripts/blast.pl blastp cog ../proteins.fasta 1e-5 80 cog 5

../../../scripts/cog_from_xml.pl cog.xml

../../../scripts/cog_R.pl cog_cog_class_annot.xls cog morchella_importuna_M04M26

../../../scripts/blast.pl blastp kog ../proteins.fasta 1e-5 80 kog 5

../../../scripts/kog_from_xml.pl kog.xml

../../../scripts/kog_R.pl kog_kog_class_annot.xls kog morchella_importuna_M04M26

cd ..

mkdir CDD

cd CDD

../../../scripts/split_fasta_file_averagely.pl ../proteins.fasta 3

\# 将序列提交到https://www.ncbi.nlm.nih.gov/Structure/bwrpsb/bwrpsb.cgi

\# QM3-qcdsearch-12F646D4E3484E1D

\# QM3-qcdsearch-1869529046994975

\# QM3-qcdsearch-335040FC0118F61B

\# mi0_hitdata.txt

cat *.txt | perl -e 'while (<>) { if (m/^#/ or m/^\s*$/) { next; } elsif (m/^Query/) { print} else { s/.*?>//; $out{$_} = 1; } } foreach (sort keys %out) { print } ' | uniq > CDD.tab

rm *.fasta *.txt

cd ..

mkdir interpro

cd interpro

../../../scripts/interProScan5.pl ../proteins.fasta chenllianfu@gmail.com interpro5 30

cd ..

mkdir go

cd go

\# b2g4pipe

cd b2g4pipe

../../../../scripts/split_blast_xml.pl ../../nr/nr.xml 20

for i in `ls ??.xml`

do

i=${i/.xml/}

echo "java -cp *:ext/*: es.blast2go.prog.B2GAnnotPipe -in $i.xml -out go_$i -annot -dat -annex"

done > command.blast2go.list

ParaFly -c command.blast2go.list -CPU 20

cd ..

cat b2g4pipe/go_??.annot | grep GO | cut -f 1,2 > go.annot

../../../scripts/merge_interpro_to_go.pl go.annot ../interpro/interpro5.tsv > aa; mv aa go.annot

cd ..

mkdir kaas

cd kaas

\# 在 http://www.genome.jp/kaas-bin/kaas_main 网页工具中提交序列进行注释

#wget http://www.genome.jp/tools/kaas/files/dl/1522086213/query.ko

cd ..

mkdir tf

cd tf

../../../scripts/interpro2tf.pl ../interpro/interpro5.tsv

cd ..
