# Supplementary material for: Opposite Polarity Monospore Genome De Novo Sequencing and Comparative Analysis Reveal the Possible Heterothallic Life Cycle of Morchella importuna
Source: Int J Mol Sci. 2018 Aug 25;19(9):2525. doi: 10.3390/ijms19092525 (PMC6164635; doi:10.3390/ijms19092525)
Supplement: Supplementary file 1 [file ijms-19-02525-s001.zip › Supplement/Supplementary Material 3.doc]

mkdir 0.initial_data

cd 0.initial_data

ln -s ../../2018-03-06_genome_analysis_of_morchella_importuna_M04M24/1.genome_denovo_assembling/genome.fasta genome.M04M24.fasta

ln -s ../../2018-03-23_genome_analysis_of_morchella_importuna_M04M26/1.genome_denovo_assembling/Morchella_importuna_v1.0_GS.fasta genome.M04M26.fasta

ln -s /share/disk01_28T/chenlianfu/2018-03-06_genome_analysis_of_morchella_importuna_M04M24/2.genome_annotation/d.functional_annotation/proteins.fasta proteins.MIM04M24.fasta

ln -s /share/disk01_28T/chenlianfu/2018-03-23_genome_analysis_of_morchella_importuna_M04M26/2.genome_annotation/d.functional_annotation/proteins.fasta proteins.MIM04M26.fasta

ln -s ../../2018-03-06_genome_analysis_of_morchella_importuna_M04M24/2.genome_annotation/c.protein_coding_gene_prediction/genome.gff3 genome.M04M24.gff3

ln -s ../../2018-03-23_genome_analysis_of_morchella_importuna_M04M26/2.genome_annotation/c.protein_coding_gene_prediction/genome.gff3 genome.M04M26.gff3

ln -s ../../2018-03-06_genome_analysis_of_morchella_importuna_M04M26/2.genome_annotation/d.functional_annotation/go/go.annot go.M04M26.annot

ln -s ../../2018-03-23_genome_analysis_of_morchella_importuna_M04M26/2.genome_annotation/d.functional_annotation/go/go.annot go.M04M26.annot

ln -s ../../2018-03-06_genome_analysis_of_morchella_importuna_M04M24/2.genome_annotation/d.functional_annotation/kaas/query.ko query.M04M24.ko

ln -s ../../2018-03-23_genome_analysis_of_morchella_importuna_M04M26/2.genome_annotation/d.functional_annotation/kaas/query.ko query.M04M26.ko

mkdir homolog_proteins

cd homolog_proteins

\# 加入多个真菌物种的全基因组蛋白fasta文件

cd ..

cd ..

mkdir 1.orthomcl_core_pan

cd 1.orthomcl_core_pan

echo "CREATE DATABASE IF NOT EXISTS orthomcl_chenlianfu_20180327" | mysql -utrain -p123456

perl -p -e 's/:3307/_chenlianfu_20180327/; s/^dbLogin=.*/dbLogin=train/; s/^dbPassword=.*/dbPassword=123456/' /opt/biosoft/orthomclSoftware-v2.0.9/doc/OrthoMCLEngine/Main/orthomcl.config.template > orthomcl.config

orthomclInstallSchema orthomcl.config

mkdir compliantFasta

cd compliantFasta

orthomclAdjustFasta M04M24 ../../0.initial_data/proteins.MIM04M24.fasta 1

orthomclAdjustFasta M04M26 ../../0.initial_data/proteins.MIM04M26.fasta 1

cd ..

orthomclFilterFasta compliantFasta/ 20 20

makeblastdb -in goodProteins.fasta -dbtype prot -title orthomcl -parse_seqids -out orthomcl -logfile orthomcl.log

blast.pl blastp orthomcl goodProteins.fasta 1e-5 4 blastResult 6

orthomclBlastParser blastResult.tab compliantFasta/ > similarSequences.txt

perl -p -i -e 's/0\t0/1\t-181/' similarSequences.txt

orthomclLoadBlast orthomcl.config similarSequences.txt

orthomclPairs orthomcl.config orthomcl_pairs.log cleanup=no

orthomclDumpPairsFiles orthomcl.config

mcl mclInput --abc -I 1.5 -o mclOutput

orthomclMclToGroups OCG 1 < mclOutput > groups.txt

orthomcl_genes_number_stats.pl groups.txt compliantFasta > genes_number_stats.txt

mkdir GO_class_of_unique_genes

cd GO_class_of_unique_genes

head -n 2 ../genes_number_stats.txt | tail -n 1 | cut -f 3 | perl -p -i -e 's/\s+/\n/g' | sort > unique_gene.M04M24.list

perl -e 'open IN, "unique_gene.M04M24.list"; while (<IN>) { chomp; $gene{$_} = 1; } while (<>) { if (m/^>(\w+)/) { if (exists $gene{$1}) { $keep = 1; } else { $keep = 0; } } print if $keep == 1; }' ../../0.initial_data/gene.M04M24.fasta > unique_gene.M04M24.fasta

blast.pl blastn MIM04M26.genome unique_gene.M04M24.fasta 1e-9 80 M04M24_VS_M04M26 5

parsing_blast_result.pl M04M24_VS_M04M26.xml 0 1e-9 1 > M04M24_VS_M04M26.tab

perl -e '<>; while (<>) { @_ = split /\t/; print "$_[0]\n" if $_[8] == 1; }' M04M24_VS_M04M26.tab > M04M24_VS_M04M26.error

diff2file.pl M04M24_VS_M04M26.error unique_gene.M04M24.list --out

mv file2_only unique_gene.M04M24.list

rm file*

head -n 6 ../genes_number_stats.txt | tail -n 1 | cut -f 3 | perl -p -i -e 's/\s+/\n/g' | sort > unique_gene.M04M26.list

perl -e 'open IN, "unique_gene.M04M26.list"; while (<IN>) { chomp; $gene{$_} = 1; } while (<>) { if (m/^>(\w+)/) { if (exists $gene{$1}) { $keep = 1; } else { $keep = 0; } } print if $keep == 1; }' ../../0.initial_data/gene.M04M26.fasta > unique_gene.M04M26.fasta

blast.pl blastn MIM04M24.genome unique_gene.M04M26.fasta 1e-9 80 M04M26_VS_M04M24 5

parsing_blast_result.pl M04M26_VS_M04M24.xml 0 1e-9 1 > M04M26_VS_M04M24.tab

perl -e '<>; while (<>) { @_ = split /\t/; print "$_[0]\n" if $_[8] == 1; }' M04M26_VS_M04M24.tab > M04M26_VS_M04M24.error

diff2file.pl M04M26_VS_M04M24.error unique_gene.M04M26.list --out

mv file2_only unique_gene.M04M26.list

rm file*

/opt/biosoft/go_class/bin/annot2wego.pl ../../0.initial_data/go.M04M24.annot > go.M04M24.wego

/opt/biosoft/go_class/bin/annot2wego.pl ../../0.initial_data/go.M04M26.annot > go.M04M26.wego

perl -e 'open IN, "unique_gene.M04M24.list"; while (<IN>) { chomp; $gene{$_} = 1; } while (<>) { print if (m/^(\S+)/ && exists $gene{$1}) }' go.M04M24.wego > aa; mv aa go.M04M24.wego

perl -e 'open IN, "unique_gene.M04M26.list"; while (<IN>) { chomp; $gene{$_} = 1; } while (<>) { print if (m/^(\S+)/ && exists $gene{$1}) }' go.M04M26.wego > aa; mv aa go.M04M26.wego

/opt/biosoft/go_class/bin/go_svg.pl --name out --color "red:green" --mark "M04M24:M04M26" --note "GO Class of Unique Genes" go.M04M24.wego go.M04M26.wego

#perl -p -i -e 's/MovePer:.*/MovePer:0.35/; s/FontSize:.*/FontSize:12/; s/$/ /' out.lst

perl -p -i -e 's/MovePer:.*/MovePer:0.35/; s/FontSize:.*/FontSize:16/; s/$//' out.lst

/opt/biosoft/go_class/svg/distributing_svg.pl out.lst out.svg

/opt/biosoft/go_class/bin/changsvgsize.pl out.svg 200 -150

convert out.svg out.png

cd ..

mkdir GO_enrichment_analysis

cd GO_enrichment_analysis

/opt/biosoft/go_class/bin/annot2gaf.pl /opt/biosoft/go_class/bin/gene_ontology_edit.obo ../../0.initial_data/go.M04M24.annot > gene_association.M04M24.gaf2

/opt/biosoft/go_class/bin/annot2gaf.pl /opt/biosoft/go_class/bin/gene_ontology_edit.obo ../../0.initial_data/go.M04M26.annot > gene_association.M04M26.gaf2

ln -s ../GO_class_of_unique_genes/unique_gene.*.list .

\# /opt/sysoft/jre1.7.0_05/bin/javaws http://compbio.charite.de/tl_files/ontologizer/webstart/ontologizer.jnlp

cd ..

mkdir kaas_enrichment_analysis

cd kaas_enrichment_analysis

../../scripts/kaas2pathwayEnrichmentAnalysis.pl ../../0.initial_data/query.M04M24.ko ../GO_enrichment_analysis/unique_gene.M04M24.list

../../scripts/kaas2pathwayEnrichmentAnalysis.pl ../../0.initial_data/query.M04M26.ko ../GO_enrichment_analysis/unique_gene.M04M26.list

cd ..

cd ..

mkdir 2.orthomcl_phylogeny

cd 2.orthomcl_phylogeny

mkdir proteins

cd proteins

../../scripts/format_protein_fasta.pl ../../0.initial_data/proteins.MIM04M24.fasta mim24

../../scripts/format_protein_fasta.pl ../../0.initial_data/proteins.MIM04M26.fasta mim26

for i in `ls ../../0.initial_data/homolog_proteins/*.fasta`

do

x=`perl -e '$name = $1 if $ARGV[0] =~ m/homolog_proteins\/(\w{5})/; $name =~ s/(\w)/\L$1/; print $name;' $i`

../../scripts/format_protein_fasta.pl $i $x

perl -p -i -e 's/>jgi\|(\w+)\|(\w+)\|.*/>${1}_$2/' $x.fasta

done

cd ..

mkdir compliantFasta

cd compliantFasta

for i in `ls ../proteins/*.fasta`

do

x=${i/*\//}

x=${x/.fasta/}

echo "orthomclAdjustFasta $x $i 1"

done | sh

cd ..

echo "CREATE DATABASE IF NOT EXISTS orthomcl_chenlianfu_20180402" | mysql -utrain -p123456

perl -p -e 's/orthomcl:3307/orthomcl_chenlianfu_20180402/; s/^dbLogin=.*/dbLogin=train/; s/^dbPassword=.*/dbPassword=123456/' /opt/biosoft/orthomclSoftware-v2.0.9/doc/OrthoMCLEngine/Main/orthomcl.config.template > orthomcl.config

orthomclInstallSchema orthomcl.config

orthomclFilterFasta compliantFasta/ 30 20

makeblastdb -in goodProteins.fasta -dbtype prot -title orthomcl -parse_seqids -out orthomcl -logfile orthomcl.log

../scripts/blast.pl blastp orthomcl goodProteins.fasta 1e-5 24 blastResult 6

orthomclBlastParser blastResult.tab compliantFasta/ > similarSequences.txt

perl -p -i -e 's/0\t0/1\t-181/' similarSequences.txt

orthomclLoadBlast orthomcl.config similarSequences.txt

orthomclPairs orthomcl.config orthomcl_pairs.log cleanup=no

orthomclDumpPairsFiles orthomcl.config

mcl mclInput --abc -I 1.5 -o mclOutput

orthomclMclToGroups OCG 1 < mclOutput > groups.txt

orthomcl_genes_number_stats.pl groups.txt compliantFasta > genes_number_stats.txt

mkdir species_tree

cd species_tree

../../scripts/orthomcl_findSingleCopyOrtholog.pl ../groups.txt ../compliantFasta/

../../scripts/fastas2aligned_by_mafft.pl SingleCopyOrthologGroups 24

Gblocks allSingleCopyOrthologsAlign.fasta -t=p

/opt/biosoft/RAxML_v8.1.24/usefulScripts/convertFasta2Phylip.sh allSingleCopyOrthologsAlign.fasta-gb > allSingleCopyOrthologsAlign.phy

java -jar /opt/biosoft/prottest-3.4-20140123/prottest-3.4.jar -i allSingleCopyOrthologsAlign.phy -all-distributions -F -AIC -BIC -tc 0.5 -threads 6 -o prottest.out

mkdir RAxML

cd RAxML

raxmlHPC-HYBRID-SSE3 -f a -x 12345 -p 12345 -# 1000 -m PROTGAMMAILGX -s ../allSingleCopyOrthologsAlign.phy -n out -T 24

\# java -jar /opt/biosoft/FigTree_v1.4.2/lib/figtree.jar RAxML_bipartitions.out

cd ../..

mkdir r8s

cd r8s

echo '#NEXUS

begin trees;

tree tree_1 = [&R] ((schpo:0.552844,((((aspni:0.207057,(xylhe:0.120387,dipse:0.191785):0.021727):0.022164,(neucr:0.213402,sclsc:0.130018):0.07056):0.093832,(artol:0.273381,(((mim24:3.6E-5,mim26:6.8E-4):0.09604,tubme:0.115337):0.0442,pyrco:0.200408):0.064274):0.030079):0.131264,sacce:0.762239):0.063317):0.121243,((pospl:0.131884,(pleos:0.134346,schco:0.183832):0.037581):0.209692,(ustma:0.355395,rhoba:0.393877):0.04868):0.121243);

end;

begin r8s;

blformat lengths=persite nsites=250814 ulrametric=no;

MRCA AGA pleos schco;

MRCA ASC aspni neucr;

fixage taxon=AGA age=162;

fixage taxon=ASC age=394;

#divtime method=PL crossv=yes fossilfixed=yes cvstart=1 cvinc=1 cvnum=10;

#divtime method=PL crossv=yes fossilfixed=yes cvstart=0 cvinc=0.5 cvnum=10;

#divtime method=PL crossv=yes fossilfixed=yes cvstart=-5 cvinc=0.5 cvnum=20;

#divtime method=PL crossv=yes fossilfixed=yes cvstart=-4 cvinc=0.2 cvnum=15;

#divtime method=PL crossv=yes cvstart=1 cvinc=1 cvnum=10;

#divtime method=PL crossv=yes cvstart=-4 cvinc=0.2 cvnum=15;

set smoothing=100000000;

divtime method=PL algorithm=TN;

showage;

describe plot=cladogram;

describe plot=chrono_description;

end;' > r8s_in.txt

r8s -b -f r8s_in.txt > r8s_out.txt

perl -e 'while (<>) { if (m/tree tree_1 = (.*);/) { my $tree = $1; $tree =~ s/(BOL|AGA|ASC)//g; print "$tree\n"; } }' r8s_out.txt > tree.txt

cd ..

mkdir cafe

cd cafe

../../scripts/orthoMCL2cafe.pl ../groups.txt > orthomcl2cafe.tab

echo '#!/opt/biosoft/cafehahnlab-code/cafe/cafe

version

date

load -i orthomcl2cafe.tab -t 4 -p 0.05

tree ((schpo:857.797809,((((aspni:340.652560,(xylhe:291.247403,dipse:291.247403):49.405158):53.347440,(neucr:277.477326,sclsc:277.477326):116.522674):129.856909,(artol:451.532383,(((mim24:0.645892,mim26:0.645892):200.490957,tubme:201.136849):105.153196,pyrco:306.290045):145.242338):72.324526):259.238425,sacce:783.095334):74.702475):140.978552,((pospl:234.221407,(pleos:162.000000,schco:162.000000):72.221407):464.616029,(ustma:615.621348,rhoba:615.621348):83.216088):299.938924)

lambda -s

report out' > cafe_command

chmod 755 cafe_command

./cafe_command > cafe.log

../../scripts/parsing_cafeOut.pl out.cafe mim24 > cafe_out.txt 2> cafe_mim24.txt

../../scripts/parsing_cafeOut.pl out.cafe mim26 > cafe_out.txt 2> cafe_mim26.txt

cd ..

mkdir 3.CAZYme

cd 3.CAZYme

mkdir a.dbCNA_blastp

cd a.dbCNA_blastp

for i in `ls ../../2.orthomcl_phylogeny/proteins/*.fasta`

do

x=${i/.fasta/}

x=${x/*\//}

echo "/opt/biosoft/dbCAN_v5.0/blast.pl blastp /opt/biosoft/dbCAN_v5.0/CAZyDB.07152016 $i 1e-5 80 $x 6"

done > command.blast.list

ParaFly -c command.blast.list -CPU 1

cd ..

mkdir b.dbCAN_hmmscan

cd b.dbCAN_hmmscan

for i in `ls ../../2.orthomcl_phylogeny/proteins/*.fasta`

do

x=${i/.fasta/}

x=${x/*\//}

echo "/opt/biosoft/dbCAN_v5.0/para_hmmscan.pl --out $x --chunk 100 --cpu 80 --hmmscan \" --cpu 1 -E 1e-3 --domE 1e-3\" --hmm_db /opt/biosoft/dbCAN_v5.0/dbCAN-fam-HMMs.txt --tmp_prefix ${x}_hmmscan $i"

done > command.para_hmmscan.list

ParaFly -c command.para_hmmscan.list -CPU 1

cd ..

mkdir c.combination

cd c.combination

cat ../a.dbCNA_blastp/*.tab > blastp.tab

cat ../b.dbCAN_hmmscan/*.domtbl > hmmscan.domtbl

cat ../../2.orthomcl_phylogeny/proteins/*.fasta > query.fasta

/opt/biosoft/dbCAN_v5.0/dbcan_combine.pl --query query.fasta --CAZy_blastDB /opt/biosoft/dbCAN_v5.0/CAZyDB.07152016.fa hmmscan.domtbl blastp.tab

cd ..

/opt/biosoft/dbCAN_v5.0/dbcan_stats.pl c.combination/out.tab ../2.orthomcl_phylogeny/proteins/ d.result

cd d.result

for i in `ls ../../2.orthomcl_phylogeny/proteins/*.fasta`

do

x=${i/.fasta/}

x=${x/*\//}

../../scripts/fasta_extract_subseqs_from_list.pl $i $x.tab > CAZyme.$x.fasta

done

cd ..

mkdir e.singalp

cd e.singalp

for i in `ls ../../2.orthomcl_phylogeny/proteins/*.fasta`

do

x=${i/.fasta/}

x=${x/*\//}

echo "signalp ../d.result/CAZyme.$x.fasta > signalp.$x.out"

done > command.signalp.list

ParaFly -c command.signalp.list -CPU 17

for i in `ls ../../2.orthomcl_phylogeny/proteins/*.fasta`

do

x=${i/.fasta/}

x=${x/*\//}

grep -P "\sY\s" signalp.$x.out > abc;

../../scripts/fasta_extract_subseqs_from_list.pl $i abc > signalp.$x.fasta

perl -e 'open IN, $ARGV[0]; <IN>; while (<IN>) { @_ = split /\t/; $annot{$_[0]}{$_[1]} = 1; } close IN; open IN, $ARGV[1]; while (<IN>) { s/^# name/name\tFamily/; @_ = split /\s+/; if (exists $annot{$_[0]}) { @annot = sort keys %{$annot{$_[0]}}; $annot = join ",", @annot; s/$_[0]/$_[0]\t$annot/; } print; }' ../d.result/$x.tab abc > signalp.$x.tab

done

rm abc

cd ..

mkdir f.NetNGlyc

cd f.NetNGlyc

for i in `ls ../../2.orthomcl_phylogeny/proteins/*.fasta`

do

x=${i/.fasta/}

x=${x/*\//}

echo "../../scripts/netNglyc_para.pl ../e.singalp/signalp.$x.fasta 80 0.5 > netNglyc.$x.out"

done > command.netNglyc_para.list

ParaFly -c command.netNglyc_para.list -CPU 1

cd ..

cd ..

mkdir 4.colinearity

cd 4.colinearity

mkdir MIM04M26_vs_Tubme

cd MIM04M26_vs_Tubme

gzip -dc ../../0.initial_data/Tubme1_GeneCatalog_genes_20111120.gff.gz | perl -e 'while (<>) { $gene = $1 if m/name \"(.*?)\"/; push @gene, $gene unless exists $info{$gene}; @_ = split /\t/; $info{$gene}{$_[3]} = 1; $info{$gene}{$_[4]} = 1; $seq{$gene} = $_[0]; } foreach (@gene) { my @pos = keys %{$info{$_}}; @pos = sort {$a <=> $b} @pos; print "$seq{$_}\t$_\t$pos[0]\t$pos[-1]\n"; }' > tubme.gff

sort -k 1.10n -k 3n tubme.gff > 11; mv 11 tubme.gff

gzip -dc ../../0.initial_data/Tubme1_GeneCatalog_proteins_20111120.aa.fasta.gz | perl -p -e 's/^>.*\|/>/; s/\*$//' > tubme.fasta

perl -e 'while (<>) { if (m/^(\S+)\t.*\tgene\t(\d+)\t(\d+).*ID=([^;\s]+)/) { print "$1\t$4\t$2\t$3\n" } }' ../../0.initial_data/genome.M04M26.gff3 > morim.gff

ln -s ../../0.initial_data/proteins.MIM04M26.fasta morim.fasta

cat morim.fasta tubme.fasta > all.proteins.fasta

makeblastdb -in all.proteins.fasta -dbtype prot -title all -parse_seqids -out all -logfile all.log

../../scripts/blast.pl blastp all all.proteins.fasta 1e-5 80 blast 6

mkdir data

../../scripts/blastOut6_filter.pl blast.tab all.proteins.fasta 20.0 1e-9 0.2 > data/mi_tm.blast

cat morim.gff tubme.gff > data/mi_tm.gff

MCScanX data/mi_tm

grep -P "MI.*GS" data/mi_tm.collinearity > data/mi_tm.collinearity_intraspecific

../../scripts/mcscanx_stats_blocks.pl data/mi_tm.collinearity_intraspecific > data/mi_tm.collinearity_intraspecific.stats

grep -P "MI.*MI" data/mi_tm.collinearity > data/mi_tm.collinearity_intraspecific_MI

../../scripts/mcscanx_stats_blocks.pl data/mi_tm.collinearity_intraspecific_MI > data/mi_tm.collinearity_intraspecific_MI.stats

grep -P "GS.*GS" data/mi_tm.collinearity > data/mi_tm.collinearity_intraspecific_GS

../../scripts/mcscanx_stats_blocks.pl data/mi_tm.collinearity_intraspecific_GS > data/mi_tm.collinearity_intraspecific_GS.stats

cd ..

mkdir MIM04M24_vs_MIM04M26_nucmer

cd MIM04M24_vs_MIM04M26_nucmer

ln -s ../../0.initial_data/genome.M04M2*.fasta ./

nucmer --maxmatch -c 200 -g 200 genome.M04M26.fasta genome.M04M24.fasta

dnadiff -d out.delta

#mgaps -l 200 -f .12 -s 600 < out.delta > out.gaps

#combineMUMs -x -e .10 -W out.errorsgaps genome.M04M26.fasta genome.M04M24.fasta out.gaps > out.align

delta-filter -i 98 -l 2000 -1 out.delta > out.f.delta

mummerplot -f -l -p out -s large -t png out.f.delta

cd ..

mkdir MIM04M24_vs_MIM04M26_MCScanX

cd MIM04M24_vs_MIM04M26_MCScanX

perl -e 'while (<>) { if (m/^(\S+)\t.*\tgene\t(\d+)\t(\d+).*ID=([^;\s]+)/) { print "$1\t$4\t$2\t$3\n" } }' ../../0.initial_data/genome.M04M26.gff3 > M04M26.gff

perl -e 'while (<>) { if (m/^(\S+)\t.*\tgene\t(\d+)\t(\d+).*ID=([^;\s]+)/) { print "$1\t$4\t$2\t$3\n" } }' ../../0.initial_data/genome.M04M24.gff3 > M04M24.gff

ln -s ../../0.initial_data/proteins.MIM04M26.fasta M04M26.fasta

ln -s ../../0.initial_data/proteins.MIM04M24.fasta M04M24.fasta

cat M04M26.fasta M04M24.fasta > all.proteins.fasta

makeblastdb -in all.proteins.fasta -dbtype prot -title all -parse_seqids -out all -logfile all.log

../../scripts/blast.pl blastp all all.proteins.fasta 1e-5 80 blast 6

mkdir data

../../scripts/blastOut6_filter.pl blast.tab all.proteins.fasta 20.0 1e-9 0.2 > data/comp.blast

cat M04M26.gff M04M24.gff > data/comp.gff

MCScanX data/comp

mkdir 5.circos

cd 5.circos

mkdir exp_cal

cd exp_cal

mkdir M04M24

cd M04M24

ln -s ../../../../2018-03-06_genome_analysis_of_morchella_importuna_M04M24/2.genome_annotation/c.protein_coding_gene_prediction/reads.?.fastq ./

ln -s ../../../../2018-03-06_genome_analysis_of_morchella_importuna_M04M24/2.genome_annotation/genome.fasta ./

ln -s ../../../../2018-03-06_genome_analysis_of_morchella_importuna_M04M24/2.genome_annotation/c.protein_coding_gene_prediction/genome.gtf ./

hisat2_extract_splice_sites.py genome.gtf > splitSites.txt

hisat2_extract_exons.py genome.gtf > exonSites.txt

hisat2-build --ss splitSites.txt --exon exonSites.txt genome.fasta genome

hisat2 -x genome -p 80 --min-intronlen 20 --max-intronlen 4000 -1 reads.1.fastq -2 reads.2.fastq -S hisat2.sam --new-summary --summary-file hisat2.summary

htseq-count -f sam -r name -s no -a 10 -t exon -i gene_id hisat2.sam genome.gtf > rawCounts.txt

htseq_outs2matrix.pl sample rawCounts.txt > raw_counts.matrix

rawCounts2TPM.pl raw_counts.matrix genome.gtf > TPM_notCross.matrix

cd ..

mkdir M04M26

cd M04M26

ln -s ../../../../2018-03-23_genome_analysis_of_morchella_importuna_M04M26/2.genome_annotation/c.protein_coding_gene_prediction/reads.?.fastq ./

ln -s ../../../../2018-03-23_genome_analysis_of_morchella_importuna_M04M26/2.genome_annotation/genome.fasta ./

ln -s ../../../../2018-03-23_genome_analysis_of_morchella_importuna_M04M26/2.genome_annotation/c.protein_coding_gene_prediction/genome.gtf ./

hisat2_extract_splice_sites.py genome.gtf > splitSites.txt

hisat2_extract_exons.py genome.gtf > exonSites.txt

hisat2-build --ss splitSites.txt --exon exonSites.txt genome.fasta genome

hisat2 -x genome -p 80 --min-intronlen 20 --max-intronlen 4000 -1 reads.1.fastq -2 reads.2.fastq -S hisat2.sam --new-summary --summary-file hisat2.summary

htseq-count -f sam -r name -s no -a 10 -t exon -i gene_id hisat2.sam genome.gtf > rawCounts.txt

htseq_outs2matrix.pl sample rawCounts.txt > raw_counts.matrix

rawCounts2TPM.pl raw_counts.matrix genome.gtf > TPM_notCross.matrix

cd ..

cd ..

mkdir snp_INDEL

cd snp_INDEL

ln -s ../../0.initial_data/genome.M04M2*.fasta ./

bowtie2-build genome.M04M24.fasta genome.M04M24

bowtie2-build genome.M04M26.fasta genome.M04M26

ln -s ../../../2018-03-23_genome_analysis_of_morchella_importuna_M04M26/1.genome_denovo_assembling/04.FindErrors/fragment.paired.A.fastq reads26.1.fastq

ln -s ../../../2018-03-23_genome_analysis_of_morchella_importuna_M04M26/1.genome_denovo_assembling/04.FindErrors/fragment.paired.B.fastq reads26.2.fastq

ln -s ../../../2018-03-06_genome_analysis_of_morchella_importuna_M04M24/1.genome_denovo_assembling/01.trimmomatic/A.1.fastq reads24_1.1.fastq

ln -s ../../../2018-03-06_genome_analysis_of_morchella_importuna_M04M24/1.genome_denovo_assembling/01.trimmomatic/A.2.fastq reads24_1.2.fastq

ln -s ../../../2018-03-06_genome_analysis_of_morchella_importuna_M04M24/1.genome_denovo_assembling/01.trimmomatic/B.1.fastq reads24_2.1.fastq

ln -s ../../../2018-03-06_genome_analysis_of_morchella_importuna_M04M24/1.genome_denovo_assembling/01.trimmomatic/B.2.fastq reads24_2.2.fastq

bowtie2 -p 80 -x genome.M04M24 -I 0 -X 1200 --fr --score-min L,-0.3,-0.3 --rg-id S26 --rg SM:S26 --rg PL:ILLUMINA -1 reads26.1.fastq -2 reads26.2.fastq -S M26vsM24.sam 2> bowtie2.M26vsM24.log

bowtie2 -p 80 -x genome.M04M26 -I 0 -X 1200 --fr --score-min L,-0.3,-0.3 --rg-id S24 --rg SM:S24 --rg PL:ILLUMINA -1 reads24_1.1.fastq,reads24_2.1.fastq -2 reads24_1.2.fastq,reads24_2.2.fastq -S M24vsM26.sam 2> bowtie2.M24vsM26.log

samtools sort -@ 40 -m 2G -o M26vsM24.bam -O BAM M26vsM24.sam

samtools sort -@ 40 -m 2G -o M24vsM26.bam -O BAM M24vsM26.sam

samtools index M26vsM24.bam

samtools index M24vsM26.bam

samtools faidx genome.M04M24.fasta

samtools faidx genome.M04M26.fasta

java -jar /opt/biosoft/picard-tools-2.9.0/picard.jar CreateSequenceDictionary R=genome.M04M24.fasta O=genome.M04M24.dict

java -jar /opt/biosoft/picard-tools-2.9.0/picard.jar CreateSequenceDictionary R=genome.M04M26.fasta O=genome.M04M26.dict

gatk HaplotypeCaller -R genome.M04M26.fasta -I M24vsM26.bam -ERC NONE -O M24vsM26.vcf --genotyping-mode DISCOVERY --pcr-indel-model CONSERVATIVE --sample-ploidy 2 --min-base-quality-score 10 --kmer-size 10 --kmer-size 25

gatk VariantFiltration -R genome.M04M26.fasta -O M24vsM26.f.vcf -V M24vsM26.vcf --filter-name FilterQual --filter-expression "QUAL < 30.0" --filter-name FilterQD --filter-expression "QD < 13.0" --filter-name FilterMQ --filter-expression "MQ < 20.0" --filter-name FilterFS --filter-expression "FS > 20.0" --filter-name FilterMQRankSum --filter-expression "MQRankSum < -3.0" --filter-name FilterReadPosRankSum --filter-expression "ReadPosRankSum < -3.0"

grep -P "\tFilter" -v M24vsM26.f.vcf > M24vsM26.filter.vcf

gatk HaplotypeCaller -R genome.M04M24.fasta -I M26vsM24.bam -ERC NONE -O M26vsM24.vcf --genotyping-mode DISCOVERY --pcr-indel-model CONSERVATIVE --sample-ploidy 2 --min-base-quality-score 10 --kmer-size 10 --kmer-size 25

gatk VariantFiltration -R genome.M04M24.fasta -O M26vsM24.f.vcf -V M26vsM24.vcf --filter-name FilterQual --filter-expression "QUAL < 30.0" --filter-name FilterQD --filter-expression "QD < 13.0" --filter-name FilterMQ --filter-expression "MQ < 20.0" --filter-name FilterFS --filter-expression "FS > 20.0" --filter-name FilterMQRankSum --filter-expression "MQRankSum < -3.0" --filter-name FilterReadPosRankSum --filter-expression "ReadPosRankSum < -3.0"

grep -P "\tFilter" -v M26vsM24.f.vcf > M26vsM24.filter.vcf

perl -p -e 's/\s*#.*//; s/^\s*$//; s/P3_FILE_ID/\nP3_FILE_ID/' /opt/biosoft/Misa_Primer3/p3_settings_from_chenlianfu.txt > p3_settings_file

../../scripts/VCF_InDel_primer3.pl --CPU 80 --p3_setting_file p3_settings_file --gff3_out M24vsM26.primer.gff3 M24vsM26.filter.vcf genome.M04M26.fasta > M24vsM26.primer.out

../../scripts/VCF_InDel_primer3.pl --CPU 80 --p3_setting_file p3_settings_file --gff3_out M26vsM24.primer.gff3 M26vsM24.filter.vcf genome.M04M24.fasta > M26vsM24.primer.out

mkdir -p /opt/biosoft/snpEff/data/MIM04M26

perl -pe 's/^\s*$//' ../../../2018-03-23_genome_analysis_of_morchella_importuna_M04M26/2.genome_annotation/c.protein_coding_gene_prediction/genome.bestGeneModels.gtf > /opt/biosoft/snpEff/data/MIM04M26/genes.gtf

cp ../../0.initial_data/genome.M04M26.fasta /opt/biosoft/snpEff/data/MIM04M26/sequences.fa

echo "MIM04M26.genome : morchella importuna" >> /opt/biosoft/snpEff/snpEff.config

/opt/sysoft/jre1.8.0_45/bin/java -jar /opt/biosoft/snpEff/snpEff.jar build -c /opt/biosoft/snpEff/snpEff.config -gtf22 -v MIM04M26

mkdir snpeff_M04M26

cd snpeff_M04M26

/opt/sysoft/jre1.8.0_45/bin/java -Xmx2G -jar /opt/biosoft/snpEff/snpEff.jar eff -csvStats variants.SnpEff.csv -s variants.SnpEff.html -c /opt/biosoft/snpEff/snpEff.config -v -ud 500 MIM04M26 ../M24vsM26.filter.vcf > M24vsM26.SnpEff.vcf

cd ..

cd ..

../scripts/circos_create_karyotype_by_genome.pl ../0.initial_data/genome.M04M26.fasta > karyotype.txt

../scripts/circos_create_karyotype_by_genome.pl ../0.initial_data/genome.M04M24.fasta >> karyotype.txt

../scripts/VCF_get_variants_density_for_circos.pl snp_INDEL/M24vsM26.filter.vcf snp_INDEL/genome.M04M26.fasta 100000 > variant_density.histogram_for_circos.txt

../scripts/VCF_get_variants_density_for_circos.pl snp_INDEL/M26vsM24.filter.vcf snp_INDEL/genome.M04M24.fasta 100000 >> variant_density.histogram_for_circos.txt

../scripts/circos_create_gc_histogram.pl snp_INDEL/genome.M04M26.fasta 100000 > gc.histogram.txt

../scripts/circos_create_gc_histogram.pl snp_INDEL/genome.M04M24.fasta 100000 >> gc.histogram.txt

../scripts/circos_create_expression_heatmap.pl exp_cal/M04M24/TPM_notCross.matrix ../0.initial_data/genome.M04M24.gff3 > gene_expression.heatmap.txt

../scripts/circos_create_expression_heatmap.pl exp_cal/M04M26/TPM_notCross.matrix ../0.initial_data/genome.M04M26.gff3 >> gene_expression.heatmap.txt

../scripts/circos_create_links_from_MCScanX.pl --only_calculate_interspecies ../4.colinearity/MIM04M24_vs_MIM04M26_MCScanX/data/comp.collinearity ../4.colinearity/MIM04M24_vs_MIM04M26_MCScanX/data/comp.gff > links.txt

../scripts/circos_create_repeat_heatmap_data.pl ../../2018-03-23_genome_analysis_of_morchella_importuna_M04M26/2.genome_annotation/a.repeat_analysis/genome.repeat.gff3 > plot_heatmap_repeat.txt

../scripts/circos_create_repeat_heatmap_data.pl ../../2018-03-06_genome_analysis_of_morchella_importuna_M04M24/2.genome_annotation/a.repeat_analysis/genome.repeat.gff3 >> plot_heatmap_repeat.txt

grep id=DNA plot_heatmap_repeat.txt > plot_heatmap_DNA.txt

grep id=LTR plot_heatmap_repeat.txt > plot_heatmap_LTR.txt

grep id=LINE plot_heatmap_repeat.txt >> plot_heatmap_LINE.txt

grep id=SINE plot_heatmap_repeat.txt > plot_heatmap_SINE.txt

../scripts/circos_create_multi_links_conf.pl --color end --link_radius 0.54 links.txt karyotype.txt > links.conf

\# preparing configuration files.

circos -noparanoid -conf circos.conf
